# An AT-hook transcription factor promotes transcription of histone, spliced-leader, and piRNA clusters

**DOI:** 10.1093/nar/gkaf079

**Published:** 2025-02-13

**Authors:** Yi-Hui Wang, Hannah L Hertz, Benjamin Pastore, Wen Tang

**Affiliations:** Department of Biological Chemistry and Pharmacology, The Ohio State University, Columbus, OH 43210, United States; Center for RNA Biology, The Ohio State University, Columbus, OH 43210, United States; Ohio State Biochemistry Program, The Ohio State University, Columbus, OH 43210, United States; Department of Biological Chemistry and Pharmacology, The Ohio State University, Columbus, OH 43210, United States; Center for RNA Biology, The Ohio State University, Columbus, OH 43210, United States; Department of Biological Chemistry and Pharmacology, The Ohio State University, Columbus, OH 43210, United States; Center for RNA Biology, The Ohio State University, Columbus, OH 43210, United States; Ohio State Biochemistry Program, The Ohio State University, Columbus, OH 43210, United States; Department of Biological Chemistry and Pharmacology, The Ohio State University, Columbus, OH 43210, United States; Center for RNA Biology, The Ohio State University, Columbus, OH 43210, United States; Ohio State Biochemistry Program, The Ohio State University, Columbus, OH 43210, United States

## Abstract

In all three domains of life, genes with related functions can be organized into specific genomic regions known as gene clusters. In eukaryotes, histone, piRNA (Piwi-interacting RNA), and rDNA (ribosomal DNA) clusters are among the most notable clusters which play fundamental roles in chromatin formation, genome integrity, and translation, respectively. These clusters have long been thought to be regulated by distinct transcriptional mechanisms. In this study, using *Caenorhabditis elegans* as a model system we identify ATTF-6, a member of the AT-hook family, as a key factor for the expression of histone, piRNA, and 5S rDNA-SL1 (spliced leader 1) clusters. ATTF-6 is essential for *C. elegans* viability. It forms distinct nuclear foci at both piRNA and 5S rDNA-SL1 clusters. Loss of ATTF-6 leads to a depletion of histone mRNAs, SL1 transcripts, and piRNAs. Additionally, we demonstrate that ATTF-6 is required for the recruitment of USTC (Upstream Sequence Transcription Complex) to piRNA clusters, which is necessary for piRNA production. Collectively, our findings reveal a unifying role for an AT-hook transcription factor in promoting the expression of fundamental gene clusters.

## Graphical Abstract

**Graphical Abstract:**
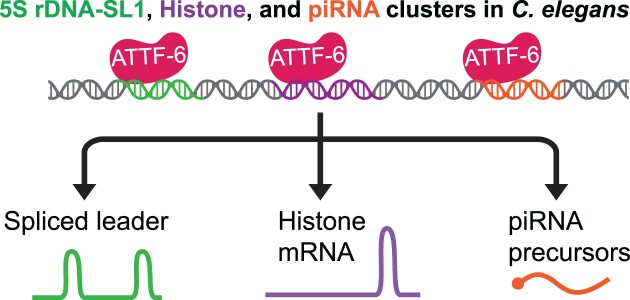

## Introduction

Genes are not randomly distributed across genomes. Instead, many genes with shared functions are organized into clusters. This organizational pattern reflects the selective pressure for co-regulation and coordinated expression of functionally related genes [1, 2]. Various types of gene clusters have been discovered. For example, CRISPR clusters provides adaptive immunity against phage infections and plasmid invasions in bacteria and archaea [3]. In eukaryotes, essential gene clusters include histone, ribosomal DNA (rDNA), and piRNA clusters, each playing essential but distinct roles in maintaining cellular homeostasis and viability. It is widely accepted that these gene clusters are subject to unique chromatin organization and transcriptional regulation that ensures the coordinated expression of their constituent genes.

Histone clusters encode histones, which serve as the fundamental building blocks of chromatin [4]. Metazoans possess replication-dependent and replication-independent histone genes. In flies and mammals, many replication-dependent histone genes are concentrated within the histone locus body, a nuclear body enriched with factors necessary for histone gene transcription and histone messenger RNA (mRNA) processing [5]. This structural arrangement creates a microenvironment conducive to the coordinated transcription of replication-dependent genes during cell cycles. Replication-dependent histone mRNAs typically lack introns and feature a conserved RNA hairpin structure in their 3′ untranslated region [4]. Unlike flies and mammals whose histone genes are tandemly arrayed, *Caenorhabditis elegans* has individual replication-dependent histone genes in small clusters dispersed throughout the genome [6]. Notably, *C. elegans* does not seem to form the histone locus body [5]. The mechanisms governing the transcription of histone genes in worms remain largely unknown.

The rDNA clusters are crucial for the synthesis of ribosomal RNA (rRNA) and subsequent assembly of ribosomes [7]. The metazoan ribosome comprises four types of rRNAs: 5S, 5.8S, 18S, and 26S. To generate ample rRNAs, rDNA genes exist in multiple copies and are typically arranged in tandem repeats [7]. In *C. elegans*, 5.8S, 18S, and 26S rRNAs are transcribed by RNA Pol I (polymerase I) from 45S rDNA loci on chromosome I [8]. One the other hand, the 5S rRNAs are transcribed separately by RNA Pol III (polymerase III) from over 100 copies of a ∼1 kb tandem repeat on chromosome V [8, 9]. Each 5S rDNA unit pairs with a spliced leader gene termed SL1 which is transcribed RNA Pol II (polymerase II) [10]. SL1 represents the major spliced leader in *C. elegans*. It is predominantly trans-spliced to the 5′ ends of many pre-mRNAs and plays an important role in translation initiation [11]. Thus far, our understanding of the transcriptional program of 5S rDNA-SL1 clusters remains limited.

Metazoan piRNA clusters produce abundant piRNAs that protect the genome against transposable elements [12]. Two different types of piRNAs have been discovered in *C. elegans* [13]. Type I piRNAs are mainly transcribed from two large clusters located on Chromosome IV [14]. The promoters of type I piRNA genes feature an 8-nt consensus sequence (CTGTTTCA), referred to as the Ruby motif flanked by upstream and downstream AT-rich sequences [14–16]. While USTC (Upstream Sequence Transcription Complex), including TOFU-4, TOFU-5, PRDE-1, and SNPC-4, associate with the Ruby motif [17–20], the binding partners and function of AT-rich sequences in piRNA transcription are completely unknown. On the other hand, type II piRNA genes share transcription start sites with actively transcribed genes throughout the genome and often do not possess the Ruby motif [13]. Both type I and type II piRNAs originate from csRNA (capped small RNA) precursors [13]. The csRNAs undergo nucleolytic processing at their 5′ and 3′ termini [21, 22], ultimately giving rise to mature piRNAs that exhibit 5′ U (uridine) and a primary length of 21-nts (nucleotides) referred to as 21U-RNAs, and are loaded to the Piwi protein termed PRG-1 [14–16, 23].

In this study, we employed a proximity-based labeling method combined with mass spectrometry to identify the proteins associated with piRNA clusters. This proteomic approach successfully captured nearly all known piRNA chromatin factors and identified several novel protein components including ATTF-6. ATTF-6 belongs to the AT-hook family which contains a core sequence, glycine–arginine–proline, known to bind to the minor groove of AT-rich DNA sequences [24]. We show that ATTF-6 forms distinct nuclear foci at piRNA and 5S rDNA-SL1 clusters. ChIP-seq (chromatin immunoprecipitation followed by high-throughput sequencing) revealed the binding of ATTF-6 to piRNA, 5S rDNA-SL1, and histone clusters. Furthermore, ATTF-6 is required for the accumulation of type I csRNAs, type I mature piRNAs, as well as SL1 and histone transcripts. Mechanistically, ATTF-6 may associate with the AT-rich sequences of piRNA promoter region and facilitate the recruitment of USTC at piRNA clusters. Taken together, our data define a crucial role of an AT-hook protein in promoting the expression of SL1, histone, and piRNAs.

## Materials and methods

### Maintenance of *C. elegans* strains

N2 strain is the reference strain for *C. elegans*. Worms were maintained under standard conditions at 20°C [25]. All animals were fed *Escherichia coli* OP50 and grown on Nematode Growth Media (NGM) unless otherwise indicated. A complete list of strains used in this study can be found in Supplementary Table S1.

### CRISPR/Cas9 genome editing

CRISPR/Cas9 genome editing in *C. elegans* was performed as previously described [26]. The TurboID::PRDE-1 and ATTF-6 degron strains were generated using TurboID and GFP::AID double-stranded donors in the N2 and mex-5p::tir1 background strain, respectively [27]. The *attf-6::3xflag* strain was created by introducing 3xFLAG at the C-terminus of ATTF-6 using a single-stranded donor. Mutations in the AT sequence at the piRNA promoter were edited with a G/C (Guanine/Cytosine)-rich single-stranded donor. Donors were incubated with a pre-assembled Cas9 complex consisting of Cas9, gRNA (guide RNA), and tracrRNA (trans-activating CRISPR RNA) (IDT). The pRF4 plasmid, containing the dominant allele of *rol-6*, was used as a co-injection marker [26]. F1 rollers were selected and screened by polymerase chain reaction (PCR). DNA donor, gRNA, and genotyping primer sequences can be found in Supplementary Table S2.

### TurboID proximity labeling and on beads digestion

A total of 40 000 synchronized TurboID::PRDE-1 and N2 L1 animals were grown at 20°C until they reached the adult stage. Animals were collected and washed twice with M9 buffer, once in ddH_2_O and once in RIPA (RNA Immunoprecipitation assay) buffer [50 mM Tris–HCl (pH 7.5), 150 mM NaCl, 0.125% SDS (sodium dodecyl sulphate), 0.125% sodium deoxycholate, 1% Triton X-100]. Animals were resuspended in RIPA buffer supplemented with cOmplete mini EDTA (ethylenediaminetetraacetic acid)-free Protease Inhibitor Cocktail tablets (Sigma–Aldrich). Worm pellets were lysed using a bead mill homogenizer (Thermo Fisher Scientific). Lysate was centrifuged and the supernatant was collected, mixed with 80 μl streptavidin magnetic beads (Thermo Fisher Scientific), and incubated overnight at 4°C with constant rotation. Beads were then washed twice with RIPA lysis buffer, once with 1 M KCl, once with 0.1 M Na_2_CO_3_, once with 2 M urea in 10 mM Tris–HCl (pH 8.0), twice again with RIPA lysis buffer, and three times with phosphate-buffered saline (PBS). Beads were resuspended in PBS for subsequent on-beads trypsin digestion.

Streptavidin magnetic beads were washed three times with 50 mM ammonium bicarbonate. Dithiothreitol (Thermo Fisher Scientific) was added, and the sample was incubated at 4°C for 15 min. Following incubation, iodoacetamide was added and the sample was kept in the dark at room temperature for 30 min. A total of 250 ng of sequencing grade-modified trypsin (Promega) in 50 mM ammonium bicarbonate was added to sample and incubated overnight at 37°C. The reaction was quenched by adding acetic acid. The supernatant was collected and concentrated for mass spectrometry analysis.

### Mass spectrometry analysis

Capillary-liquid chromatography-nanospray tandem mass spectrometry was conducted using an Orbitrap Fusion mass spectrometer equipped with an EASY-Spray source (Thermo Fisher Scientific). The MS (Mass spectrometry) data sequences were processed by converting raw files into merged MGF files using MSConvert (ProteoWizard). During data conversion, isotope distributions of the precursor ions from the MS/MS spectra were deconvoluted to determine the charge states and monoisotopic mass/charge ratios. The resulting MGF files were analyzed using Mascot Daemon (Matrix Science, version 2.5.1), and subsequently searched against the *C. elegans* Uniprot database.

### Auxin treatment

The auxin treatment procedure was carried out as previously described [28]. Briefly, the *gfp::tev::aid::attf-6; mex-5p::TIR1* strain was plated on NGM plates containing 4 mM natural auxin indole-3-acetic acid (IAA) at the L4 stage. Protein depletion was confirmed by imaging GFP (green fluorescent protein) signals in young adults. For RNA extraction experiments, 60 000 synchronized L1 larvae were plated on 150 mm NGM plates with 1 ml of concentrated OP50. Once they reached the L4 stage, all animals were collected, washed with M9 buffer, and transferred to 150 mm NGM plates containing 4 mM IAA. After reaching adulthood, worms were collected for RNA extraction.

### RNA interference by feeding of double stranded RNA

HT115 RNAi feeding bacteria were streaked from the *C. elegans* Ahringer RNAi library [29]. NGM plates containing 50 μg/ml ampicillin and 5 mM IPTG were seeded with HT115 bacteria expressing double-stranded RNA targeting the gene of interest. For the histone reporter assay, L4 larvae were placed on *attf-6* RNAi, and germlines of F1 young adults were imaged.

To explore the co-dependency between piRNA transcription factors and ATTF-6, L4 larvae of the *gfp::tev::aid::attf-6; mCherry::prde-1* strain were plated on *tofu-5* RNAi plates, and the germlines of F1 young adults were imaged. Since the RNAi effect of *snpc-4* is too strong and disrupts germline formation, L1 larvae were plated on *snpc-4* RNAi plates, and the young adult germlines of the same generation were imaged. L4440 RNAi served as the control for the all the RNAi experiments.

### Total RNA isolation and small RNA enrichment

Synchronized adult worms were collected, washed three times with the M9 buffer and once in ddH_2_O, and resuspended suspended in TRI Reagent (Sigma). Worms were lysed using the Bead Mill 24 homogenizer (Thermo Fisher Scientific) and the lysate was transferred to a clean tube. 0.1 volumes of bromochloropropane (Sigma) were added to the lysis to perform RNA extraction. Isopropanol (Sigma) was then used to precipitate RNA from the aqueous phase. Small RNAs were enriched using MirVana microRNA Isolation Kit (Thermo Fisher Scientific), according to the manufacturer’s instructions.

### Northern blotting

RNA samples were separated on 8% polyacrylamide/7 M urea gels and stained with ethidium bromide (Avantor). The samples were then transferred to Hybond-N + nylon membrane (GE Healthcare) at 400 mA for 1 h in 1 × TBE (Tris-borate-EDTA) buffer. The RNA was ultraviolet cross-linked (254 nm, 120 mJ) three times using a Stratalinker (Stratagene). Hybridizations were performed with FAM (Fluorescein amidite)-labeled anti-5S rRNA probe or FAM-labeled anti-SL1 probe (Supplementary Table S2) in ULTRAhyb buffer (Thermo Fisher Scientific) at 37°C overnight. The membrane was visualized using a Sapphire Biomolecular Imager (Azure Biosystems). The intensity of SL1 and 5S rRNA bands was measured using ImageJ.

### RT-qPCR (Reverse transcription quantitative polymerase chain reaction)

Total RNA isolated from two biologically replicated young adult samples was reverse transcribed in technical triplicates using MultiScribe Reverse Transcriptase (Thermo Fisher Scientific) with an antisense primer (Supplementary Table S2). Quantitative PCR was conducted using complementary DNA (cDNA) with PowerUp SYBR Green Master Mix (Thermo Fisher Scientific), along with gene-specific and universal primers (Supplementary Table S2), in a CFX Connect Real-Time PCR System (Bio-Rad). Relative piRNA abundances (ΔΔCt) were calculated from Ct values obtained using CFX Manager v3.1 software (Bio-Rad). Error bars represent the standard deviation (SD) in all statistical analyses.

### Small RNA sequencing library preparation

Small RNA-sequencing libraries were prepared using a previously established small RNA cloning protocol [30–32]. To sequence all types of small RNA species, small RNA samples were treated with the polyphosphatase PIR-1 to remove γ and β phosphates from 5′-triphosphorylated RNAs [32]. To clone csRNAs, small RNA samples were sequentially treated with intestinal alkaline phosphatase (NEB) and tobacco acid pyrophosphatase [13]. The monophosphorylated RNAs were ligated to a 3′ adapter (IDT, Supplementary Table S2) using T4 RNA ligase 2 (NEB) at 15°C overnight. T4 RNA ligase 1 (NEB) was used to ligate the 5′ adapter (IDT, Supplementary Table S2). The ligated products were reverse transcribed into cDNA using SuperScript IV Reverse Transcriptase (Thermo Fisher Scientific). The resulting cDNAs were amplified by PCR. Libraries with unique barcodes were pooled and sequenced on the NovaSeq platform (SP 2 × 50 bp, Illumina).

### mRNA sequencing library preparation

rRNAs were depleted using the RNase H-based method [33]. Three micrograms of total RNA was incubated with antisense DNA oligonucleotides targeting *C. elegans* and *E. coli* rRNAs, followed by treatment with Hybridase Thermostable RNase H (Biosearch Technologies) at 45°C for 30 min. The rRNA-depleted samples were treated with Turbo DNase (Thermo Fisher Scientific) at 37°C for 30 min. RNAs longer than 200 nts were enriched using RNA Clean & Concentrator-5 (Zymo Research). mRNA sequencing libraries were constructed using Ultra II Directional RNA Library Prep Kit (NEB), following the manufacturer’s instructions. The library samples, each containing unique barcodes, were pooled and sequenced on the HiSeq 4000 platform (2 × 150 bp, Illumina).

### Analysis of small RNA sequencing data

Small RNA was analyzed using our previously established small RNA pipeline [30, 31]. Raw small RNA sequencing reads were processed and low-quality reads were removed using TrimGalore [34]. The remaining reads were aligned to the reference genome (WormBase release WS279) and a reference containing annotated exon-exon junctions using Bowtie with the parameters (-v 0 -m 100 -a –best –strata) [35], then converted to BED files using BEDOPS [36]. Following alignment, each read was normalized to the total number of mapped reads and assigned to genomic features using BEDTools. Briefly, [1] reads assigned to piRNA loci were required to map uniquely and perfectly in the sense orientation, contain a 5′ T, be 15–40 nts in length, and have a 5′ end mapping to the zeroth, first, or second position of the piRNA annotation. [2] To detect piRNA precursors (csRNA), assigned reads had to map to the two nucleotides upstream of the annotated piRNA 5′ end and be 15–40 ntss in length. Comparisons between ATTF-6 depletion and the background strain were performed using custom Python scripts, and all plots were generated in R.

### Analysis of mRNA-seq data

Raw paired-end mRNA sequencing reads were removed adapters and low-quality reads using TrimGalore [34]. After adapter removal, the reads were aligned to the *C. elegans* reference genome (WormBase Release WS279) using STAR with the following parameters (-alignIntronMax 10 000 -outFilterMismatchNoverReadLmax 0.04 -outFilterIntronMotifs RemoveNoncanonical -outFilterMultimapNmax 10) [37]. Orphan reads, where only one mate in a pair aligned, were filtered out using SAMTools [38]. Transcript counts were quantified using FeatureCounts with the parameters (-p -s 2 -g gene_id -M –fraction) [39]. Genome BAM files were then converted to BigWig (BW) format using bamCoverage from the DeepTools package with the parameters (-normalize CPM -smoothLength 10 -binSize 5 -exactScaling -filterRNAstrand forward) to visualize transcript coverage [40]. Biological replicate BW files were averaged using bigWigMerge and bedGraphToBigWig from the UCSC Genome Browser. Transposon sequence levels were quantified using the Salmon tool [41]. DESeq was used to normalize read counts and calculate fold changes and *P*-values between ATTF-6 depletion and background strains [42]. The data were visualized using the R *ggplot2* package and the Integrative Genomics Viewer (IGV) genome browser [43].

### DNA-fluorescence *in situ* hybridization and immunofluorescence

The approach was adapted from an established protocol with some modifications [44, 45]. Briefly, day 1 adult worms (ATTF6::3xFlag strain) were transferred to a drop of EB (Egg Buffer) [25 mM HEPES-NaOH, pH 7.4, 118 mM NaCl, 48 mM KCl, 2 mM EDTA, 0.5 mM EGTA (ethylene glycol tetraacetic acid), with 2 mM levamisole and 0.1% Tween-20]. Gonads were dissected and fixed for 10 min by adding 4% formaldehyde in EB to reach a final concentration of 2%. After fixation, the gonads were transferred to a 1.5-ml tube and washed once with PBST (phosphate-buffered saline with Tween 20). They were then immediately immersed in pre-chilled methanol (−80°C) and incubated at room temperature for 5 min. Next, the gonads were washed twice with 2× Saline-Sodium Citrate with Tween-20, also known as SSCT (0.3 M NaCl, 0.03 M sodium citrate, pH 7.0, 0.1% Tween-20) for 5 min, and incubated in 2× SSCT with 50% formamide overnight at 37°C. For DNA probe hybridization, gonads were transferred to a new PCR tube, and 10 ng/μl of synthesized Cy3- or Cy5-labeled DNA probe (IDT) in hybridization buffer (3× SSC, 48% formamide, 10.6% dextran sulfate) was added. To denature chromosomal DNA, samples were heated at 91°C for 5 min, followed by hybridization at 37°C for 6 h in the dark. After hybridization, samples were washed three times with PBST and incubated in blocking buffer (PBST with 0.5% bovine serum albumin) at room temperature for 1 h. The anti-Flag primary antibodies (Sigma–Aldrich) were diluted 1:500 in blocking buffer and incubated with the samples overnight at 4°C. The samples were then washed three times with PBST and incubated with a goat anti-mouse Alexa 488 secondary antibody (diluted 1:500 in blocking buffer) at room temperature for 1 h in the dark. Following incubation, the samples were washed three times with PBST. Residual PBST was carefully removed, and 50 μl of DAPI (4′,6-diamidino-2-phenylindole)-containing mounting medium (Vector Laboratories) was added to each tube, followed by incubation at room temperature for 10 min. A volume of 10 μl of dissected gonads was transferred to a glass slide, covered with a glass coverslip, and sealed with nail polish.

### ChIP and sequencing library preparation

ChIP experiments were conducted as previously described [20] with minor modifications. Briefly, 60 000 worms were crosslinked in 2% formaldehyde in M9 buffer for 30 min at room temperature with constant agitation. Crosslinking was quenched by adding 125 mM glycine in M9 for 5 min at room temperature. After washing with M9 and water, the worm pellets were resuspended in FA (Formaldehyde lysis) buffer (50 mM Tris–HCl, pH 7.5, 1 mM EDTA, 1% Triton X-100, 0.1% sodium deoxycholate, 150 mM NaCl) supplemented with the protease inhibitor (Thermo). The worm pellets were then homogenized using a mixture grinder at 6 m/s for 45 s repeated three times. Next, crosslinked chromatin was sonicated for 30 cycles of 30 s ON and 30 s OFF at HIGH output. After sonication, worm lysates were centrifuged, and the supernatants were immunoprecipitated with 6 μg of anti-Flag antibody. The antibody–protein–DNA complex was pulled down using Dynabeads Protein G (Invitrogen). After washing sequentially with FA buffer, FA buffer containing 500 mM NaCl, and TE buffer (pH 8.0), reverse crosslinking was carried out by adding 1×TE (Tris-EDTA) buffer, 1% SDS, and 250 mM NaCl and incubating at 65°C for 4 h. RNA and proteins were digested by treating the samples with 20 μg of DNase-free RNase A (Invitrogen) at 37°C, followed by incubation with 200 μg of proteinase K at 60°C for 1 h. DNA was extracted using phenol-chloroform and dissolved in Tris buffer. Sequencing libraries were prepared using NEBNext Ultra II DNA Library Prep Kit (NEB), following manufacturer’s instructions.

### Analysis of ChIP-seq data and peak calling

Raw sequencing reads were pre-processed to remove adapters and poorly sequenced reads using TrimGalore [34]. Reads passing filters were then aligned to the genome using BWA-MEM using default settings [46]. BAM files from alignment were then converted to BigWig files using DeepTools bamCoverage function [40]. ChIP peaks were called using MACS2 software using the narrow peak findings option [47, 48]. Data were further analyzed using custom R and python scripts. BigWig files and called peak bed files were visualized using IGV [43].

### Quantification of ChIP-seq signals and metagene analysis

To quantify the coverage score of ChIP-seq reads across different chromosomes, the experimental BigWig files were subtracted from control BigWig files using *bigwigCompare* with the parameters (-p max –operation ratio –pseudocount 1 –binSize 5). The signals from the subtracted BigWig files were then calculated using *multiBigwigSummary* with the parameters (-bs 1000 -p max -l score –OutRawCounts). To filter out background noise, signals lower than 1.2 were removed. Subsequently, the remaining signals were grouped by chromosome, averaged across two biological replicates, and plotted using the R *ggplot2* package. ChIP-seq metagene profiles and heatmap plots were generated using the SeqPlots software [49]. BigWig files from the ChIP-seq pipeline served as input, and BED files containing type I piRNA, SL1 small nuclear RNA (snRNA), or protein-coding genes were used as references for the plots.

### Peak annotation and analysis

Since the approach used for ATTF-6 ChIP-seq differs slightly from the PRDE-1 ChIP-seq in the published study [20], different cutoff values were applied to these two datasets. To filter out noise peaks, narrow peaks identified from ATTF-6 ChIP-seq were filtered by a score of ≥200 and a significance value of ≥3. Narrow peaks from PRDE-1 ChIP-seq were filtered by a score of ≥500 and a significance value of ≥3. The filtered peaks were then assigned to annotated genes if they directly overlapped with the annotated genes or the promoter region of the annotated loci. Promoters were defined as 500 nts upstream of protein-coding gene loci and 100 nts upstream of snRNA and small nucleolar RNA (snoRNA) gene loci. Each peak was assigned to only one class if the genes were in the same direction, with priority given in the following order: snRNA promoter, snoRNA promoter, non-coding RNA (ncRNA), protein-coding gene promoter, and pseudogene. The overlap significance was assessed using a hypergeometric distribution test and visualized with a heatmap generated in R.

To construct the sequence motif of the protein binding region, genomic sequence 100 nts upstream and downstream of summit from the filtered peaks were isolated using BEDtools [50]. MEME motif analysis software was then used to generate sequence logos [51]. To evaluate the overlap between ATTF-6 and PRDE-1 peaks, enriched peaks filtered based on the aforementioned criteria were analyzed. Overlapping peaks were identified using the BEDtools intersect function, with a threshold requiring a minimum of 50% overlap between the peak regions.

### Gene ontology analysis

Protein-coding genes bound by ATTF-6 at the promoter were subjected to GO analysis using g:Profiler [52]. The results from the g:Profiler GO analysis were visualized using a custom R script.

### Microscopy

Live animals were immobilized in M9 medium containing 2 mM levamisole and mounted on fresh 4% agar pads. To prepare fixed samples, young adult worms were transferred from NGM plates into 1.5 ml tubes using M9 buffer + 0.05% Tween-20. The worms were washed three times with M9 buffer to remove bacteria, then fixed by adding pre-cooled −20°C methanol and incubated at −20°C for 5 min. Methanol was removed, and the worms were resuspended in pre-cooled −20°C acetone and incubated at −20°C for 20 min. Subsequently, acetone was replaced with PBS, and the samples were incubated at 4°C for 10 min. Residual PBS was carefully removed, and the worms were resuspended in 50 μl of DAPI-containing mounting medium (Vector Laboratories). The fixed worms were mounted onto slides by pipetting 5 μl onto the slide, covering the droplet with a coverslip, and sealing the slide with VALAP. Live animal spinning disc confocal images were acquired using MetaMorph version 7.10.4.452 on a Nikon TiE inverted microscope, equipped with an Andor Revolution WD spinning disc system and a pco.Edge bi 4.2 sCMOS detector, using a CFI Plan Apo VC 60 ×/1.2 NA water immersion objective or a 40× objective. All images from the experimental and control groups were captured using the same laser power. A maximum intensity projection (z projection) was created to visualize fluorescent signals using ImageJ. Imaging of fixed worms, DNA fluorescence *in situ* hybridization (DNA-FISH), and immunofluorescence was performed using an inverted Nikon Ti2 microscope equipped with a CrestOptics X-Light V3 spinning disc confocal module and a Hamamatsu Orca Fusion C14440-20UP detector. The system was operated by NIS Elements AR and used a Nikon CFI Plan Apo VC 60× Oil objective. Image processing was conducted in ImageJ, with z projections created to capture fluorescent signals.

### Quantification and statistical analyses

To quantify histone reporter intensity, GFP signals were measured by drawing a rectangle around nuclei and calculating the mean value within the rectangle. This process was repeated for 10 nuclei per animal, with 5 animals analyzed for each strain.

All statistical tests used in this study are listed in the figure legend. Data analysis and plotting were conducted using custom R scripts. The primary R packages utilized included *dplyr*, *ggplot2*, and other packages from the *Tidyverse* [53]. Statistical significance was determined with a *P*-value threshold of <0.05.

## Results

### Proximity labeling identifies ATTF-6, an AT-hook transcription factor that forms distinct foci in germ nuclei

**Figure 1.**
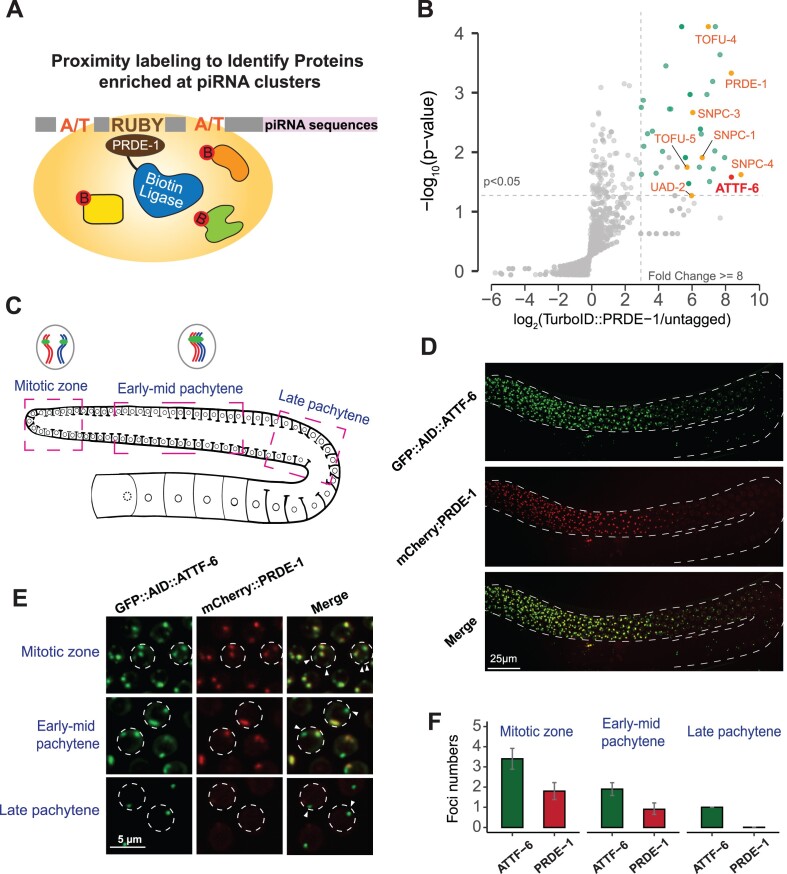
Proximity labeling identifies ATTF-6, an AT-hook transcription factor that forms distinct foci in germ nuclei. (**A**) Schematic of the proximity labeling experiment. Transcription factor PRDE-1 is tagged with a promiscuous biotin ligase (TurboID) to label proximal proteins within piRNA gene clusters. (**B**) Volcano plot showing statistically significant enriched proteins from *TurboID:: prde-1* strains [one-tailed Student’s *t*-test, *P* ≤ .05, log_2_ (fold change) ≥3, pseudocount = 0.1]. Known piRNA transcription factors and the newly identified protein, ATTF-6, is highlighted. (**C**) Schematic of the *C. elegans* gonad. The pink dashed boxes highlight the germline at different developmental stages. Chromatin and piRNA gene cluster foci formation in nuclei at various stages are depicted in gray circles. (**D**) GFP::AID::ATTF-6 and mCherry::PRDE-1 foci in the whole gonad. Confocal images (60× objective) show GFP::AID::ATTF-6 localization in the germ cells of live animals expressing mCherry::PRDE-1. The images are maximum intensity projections of a Z-stack spanning the germline. Scale bar: 25 μm. (**E**) Enlarged view of germ nuclei in the mitotic zone, early-mid pachytene, and late pachytene. Each dotted circle marks a germline nucleus. Arrowheads indicate small foci that do not colocalize with mCherry::PRDE-1 foci. Scale bar: 5 μm. (**F**) Quantification of foci numbers in germ nuclei. The numbers of GFP::AID::ATTF-6 and mCherry::PRDE-1 foci were counted in 10 nuclei from each germline zone. Error bars represent the SD.

piRNA genes constitute the largest gene family in *C. elegans* with over 15 000 discrete transcription loci that are concentrated at two mega-base long clusters [14–16]. To identify proteins associated with piRNA clusters, we employed a biotin ligase-based proximity labeling approach. Unlike conventional immunoprecipitation experiments, proximity labeling excels in capturing transient interactions and identifying interactors situated in the vicinity of the target protein [54]. We utilized TurboID, a promiscuous biotin ligase that biotinylates proximate proteins using endogenous ATP and biotin [54–56]. By applying CRISPR/Cas9 genome editing, we introduced TurboID sequences into the genomic locus of *prde-1*, which encodes a transcription factor known to localize to piRNA clusters (Fig. 1A) [17, 20]. Proteins near PRDE-1 were biotinylated *in vivo* by TurboID and then enriched using a streptavidin affinity pull-down assay [55, 56]. In brief, lysate from untagged control and the TurboID::PRDE-1 adult animals was prepared under denaturing conditions. Biotinylated proteins were pulled down using streptavidin beads under stringent washing conditions to reduce nonbiotinylated protein contaminants. The enriched proteins were then identified via mass spectrometry (Fig. 1B and Supplementary Table S3).

We found 54 proteins were significantly enriched by labeling with TurboID::PRDE-1 compared to the untagged control (normalized TurboID::PRDE-1 spectral counts ≥2, fold change ≥8, and *P*-value ≤ .05) (Fig. 1B and Supplementary Table S3). Previous genetic and biochemical studies have revealed a set of chromatin factors enriched at piRNA clusters. Strikingly, this single TurboID::PRDE-1 experiment captured nearly all previously reported chromatin factors, including TOFU-4, TOFU-5, PRDE-1, SNPC-4 collectively known as USTC [17–20], the chromodomain protein UAD-2 [57], and SNPC proteins [small nuclear RNA activating protein complex] [18, 58] (Fig. 1B). Importantly, we identified additional factors that are not been characterized in the context of the piRNA pathway. For example, TAG-72, an ortholog of human RNA cap guanine-7 methyltransferase, was enriched in the TurboID::PRDE-1 experiment (Supplementary Table S3), suggesting that the cap of piRNA precursors may be methylated.

Among newly identified proteins, ATTF-6 (AT-hook transcription factor), a member of the AT-hook family, emerged as a highly enriched candidate with a fold change of 318 (pseudocount = 0.1) (Fig. 1B). Promoters of *C. elegans* piRNA transcription loci are characterized by the Ruby motif, flanked by upstream and downstream AT-rich sequences [14, 59] (Supplementary Fig. S1A). Both Ruby and AT-rich motifs are present at piRNA-producing genes of *C. briggsae*, a sibling species that separated from *C. elegans* about 100 million years ago (Supplementary Fig. S1B) [60, 61], suggesting the promoter configuration is evolutionarily conserved among nematodes. While TOFU-4, TOFU-5, PRDE-1, and SNPC-4 associate with the Ruby motif [17–20], the binding partners and function of either upstream or downstream AT rich sequences in piRNA transcription are completely unknown. AT-hook family proteins, characterized by the presence of AT-hook motifs, preferentially bind to AT-rich regions of DNA [24, 62, 63]. AT-hook motif containing proteins, such as human high mobility group proteins, are known to play a crucial role in modulating chromatin accessibility [24, 62, 63]. The ATTF-6 protein possesses three putative AT-hook motifs, which prompted us to further explore its role in piRNA transcription.

Our study revealed that *attf-6* is an essential gene, as null alleles resulted in 100% inviable animals. Using CRISPR/Cas9 genome editing, we integrated GFP and AID (auxin-inducible degron) tags at the N-terminus of ATTF-6 which allowed us to monitor its expression and deplete ATTF-6 protein using auxin [28]. Confocal microscopy demonstrated that GFP::AID::ATTF-6 was primarily expressed in the germ nuclei in adults (Fig. 1C and D), with detectable signals in somatic cells, including muscle and intestinal cells (Supplementary Fig. S1C and D). To explore the relationship between ATTF-6 and PRDE-1, we generated a strain containing endogenously tagged *gfp::aid::attf-6* and *mCherry::prde-1* and examined ATTF-6 and PRDE-1 expression in live animals. While GFP::AID::ATTF-6 and mCherry::PRDE-1 formed foci in mitotic and meiotic nuclei, their pattern differed (Fig. 1C–F). Consistent with previous findings [17], mCherry::PRDE-1 forms two foci in mitotic nuclei, each associated with piRNA clusters at homologous chromosomes (Fig. 1C–F). Due to synapsis of homologous chromosomes (Fig. 1C), a single mCherry::PRDE-1 focus per nucleus was observed in early-mid pachytene. No mCherry::PRDE-1 foci were detected in late pachytene (Fig. 1C–F). In contrast to mCherry::PRDE-1 foci, four GFP::AID::ATTF-6 foci (two large and two small) were found in the mitotic region (Fig. 1C–F). Furthermore, there were two GFP::AID::ATTF-6 foci in early-mid pachytene and one GFP::AID::ATTF-6 focus in late pachytene (Fig. 1C–F). Notably, in both mitotic and early-mid pachytene regions, mCherry::PRDE-1 foci often co-localized with the large GFP::AID::ATTF-6 foci (Fig. 1E).

Three approaches were used to confirm the expression of PRDE-1 and ATTF-6 in the germ line. First, we fixed whole animals using methanol and paraformaldehyde and visualized nuclei using DAPI staining. The fixed gonads exhibited GFP::AID::ATTF-6 foci and mCherry::PRDE-1 foci similar to those observed in live animals (Fig. 1E and Supplementary Fig. S2A). Second, to determine whether the mCherry tag affects PRDE-1 foci formation, we generated a strain expressing GFP::PRDE-1 from its endogenous locus. GFP::PRDE-1 formed two foci in mitotic nuclei, a single focus early-mid pachytene, became undetectable in late pachytene, a pattern consistent with that of mCherry::PRDE-1 (Supplementary Fig. S2B and C). Finally, to confirm that the unique ATTF-6 foci were not due to the N-terminal tag, we examined a strain expressing ATTF-6 with a C-terminal GFP tag. Indeed, ATTF-6::GFP exhibited similar expression patterns as GFP::AID::ATTF-6 (Supplementary Fig. S2D and E). In summary, our biotin ligase-based proximity labeling approach identified ATTF-6, which forms four, two, and one nuclear foci in mitotic region, early-mid pachytene, and late pachytene, respectively.

### ATTF-6 associate with piRNA and 5S rDNA-SL1 clusters

We conducted ChIP-Seq to determine ATTF-6 binding sites across the *C. elegans* genome. Given that ATTF-6 is highly expressed in the adult germ line (Fig. 1D), we performed ChIP using the anti-Flag antibody from synchronized adults expressing endogenously tagged ATTF-6::3xFlag. ChIP-seq from untagged adults served as the negative control. ChIP-Seq experiments were conducted in biological duplicates. In parallel, we reanalyzed existing PRDE-1 ChIP-seq data alongside with its input control [20].

**Figure 2.**
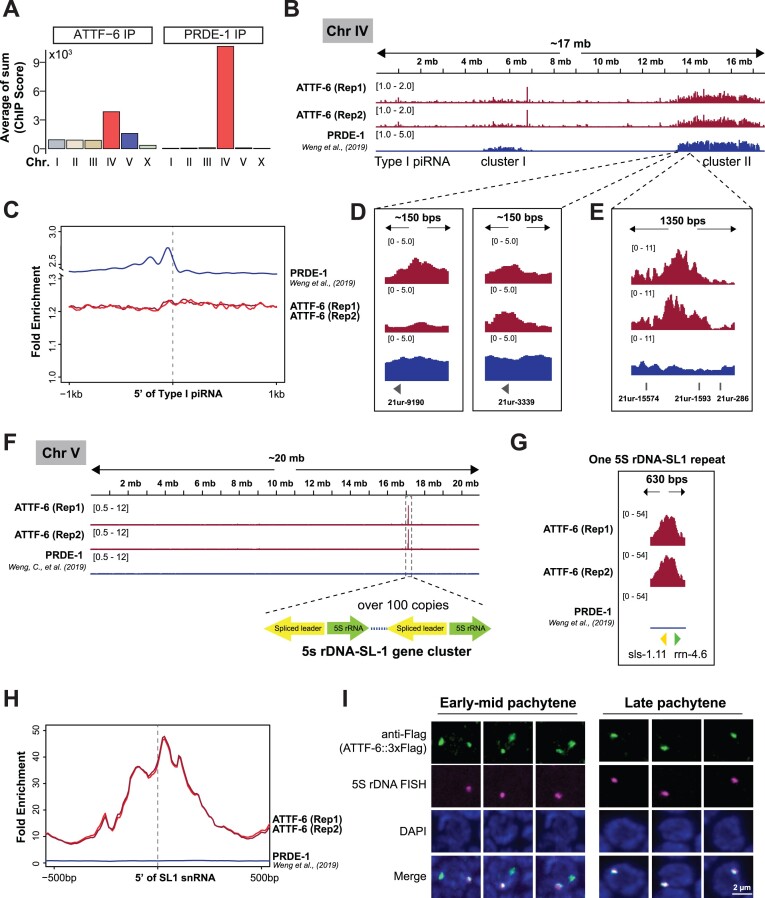
ATTF-6 associate with 5S rDNA-SL1 and piRNA clusters. (**A**) Coverage of ATTF-6 and PRDE-1 on individual chromosomes. Bar plot displaying ChIP scores summed and grouped by chromosome, showing the average score from two biological replicates. The signals were calculated in 1 kb bin. (**B**) Browser view of ChIP signals on chromosome IV. ATTF-6 and PRDE-1 ChIP-seq data were normalized to control-IP and input, respectively. Two Type I piRNA clusters on chromosome IV are labeled on the plot. ChIP-seq data for ATTF-6 from two biological replicates (Rep1 and Rep2) are shown. (**C**) Metagene profiles showing the fold enrichment of ATTF-6 and PRDE-1 at the promoters of piRNA-producing loci. The enrichment profile spans 1 kb upstream and downstream of Type I piRNA genes, aligned to the first 5′ U nucleotide of piRNAs. ChIP-seq data are shown as the average score from two biological replicates for PRDE-1 and the individual scores for ATTF-6 (Rep1 and Rep2). (**D**, **E**). Browser view zoomed in on the specific examples of ATTF-6 and PRDE-1 ChIP-Seq within piRNA clusters. (**D**) showing the promoter region of *21ur-9190* and *21ur-3339*. (**E**) displaying an unannotated gene region in the piRNA cluster. (**F**) Browser view of ChIP signals on chromosome V. ATTF-6 and PRDE-1 ChIP signals were normalized to control-IP and input. The dashed box highlights the 5S rDNA-SL-1 gene cluster, containing tandem repeats of 5S rDNA (green arrow) and SL1 (yellow arrow). ChIP-seq data for ATTF-6 from two biological replicates (Rep1 and Rep2) are shown. (**G**) Browser view zoomed in on a 5S rDNA-SL1 repeat. (**H**) Metagene profiles of ATTF-6 and PRDE-1 ChIP signals around the SL1 snRNA genes. The plot is anchored at the 5′ end of the SL1 snRNA genes and spans 500 bp upstream and downstream. ChIP-seq data are shown as the average score from two biological replicates for PRDE-1 and the individual scores for ATTF-6 (Rep1 and Rep2). (**I**) Co-localization of 5S rDNA and ATTF-6 in early-mid pachytene and late pachytene nuclei. FISH was conducted using Cy5-labeled DNA probes to visualize the 5S rDNA gene cluster, and immunostaining with an anti-Flag antibody was performed to detect ATTF-6::3xFlag protein. The confocal images (60× objective) of three germ nuclei are presented as maximum intensity projections of Z-stacks spanning the fixed germline. Scale bar: 2 μm.

When examining ChIP signals across individual chromosomes, we observed a notable enrichment of PRDE-1 on chromosome IV (Fig. 2A) [20]. In contrast, ATTF-6 exhibited a broader distribution across chromosomes, with significant enrichment on chromosomes IV and V (Fig. 2A). This global analysis was further supported by inspecting individual chromosomes. For example, both ATTF-6 and PRDE-1 were found to associate with piRNA clusters on chromosome IV (Fig. 2B). Metagene analyses demonstrated a detectable enrichment of PRDE-1 at the promoters of type I piRNA genes (Fig. 2C) [20], while ATTF-6 ChIP signals showed a modest enrichment at piRNA genes in both biological replicates (Fig. 2C and Supplementary Fig. S3A). PRDE-1 and ATTF-6 signals were also observed at the promoters of specific piRNA genes including *21ur-9190* and *21ur-3339* (Fig. 2D). Consistent with ChIP-seq data showing moderate enrichment of ATTF-6 at piRNA clusters, ChIP-qPCR revealed approximately a three-fold increase in ATTF-6 binding at the *21ur-2675* gene over the negative control (Supplementary Fig. S3B). Interestingly, some strong ATTF-6 binding sites were detected within piRNA clusters, although the peak did not overlap with any annotated piRNA genes (Fig. 2E).

On chromosome V, we identified a predominant ATTF-6 binding site which is absent in PRDE-1 ChIP data (Fig. 2F). This ATTF-6 peak coincided with the 5S rDNA-SL1 cluster (Fig. 2F). The 5S rDNA-SL1 cluster comprises over 100 tandem repeats of 5S rDNA and SL1 genes which are oriented oppositely [8, 9]. A browser view focusing on one repeat revealed that ATTF-6 was highly enriched at the promoter region of the 5S rDNA and SL1 gene (Fig. 2G). This enrichment was further supported by metagene analyses (Fig. 2H), which confirmed ATTF-6 binding to the genomic loci of 5S rDNA and SL1 genes. These findings led us to speculate that small GFP::AID::ATTF-6 foci, which did not co-localize with mCherry::PRDE-1, were associated with the 5S rDNA-SL1 locus. To test this idea, we performed 5S rDNA FISH and subsequently conducted immunofluorescence against ATTF-6::3xFlag protein using the anti-Flag antibody. Although ATTF-6::3xFlag immunofluorescence signals were poor in the mitotic region (Supplementary Fig. S3C), we consistently observed two ATTF-6 foci in early-mid pachytene nuclei, one of which co-localized with the 5S rDNA-SL1 cluster, and a single ATTF-6 focus in late pachytene nuclei that co-localized with the 5S rDNA-SL1 cluster (Fig. 2I). Furthermore, we included an additional FISH probe targeting a region (Chromosome IV: 14 987 799–15 001 823) within the piRNA cluster II [45]. In early-mid pachytene nuclei, one ATTF-6 focus co-localized with the piRNA cluster, while the other co-localized with 5S rDNA-SL1 cluster (Supplementary Fig. S3C). In late pachytene nuclei, ATTF-6 predominantly associated with the 5S rDNA-SL1 cluster (Supplementary Fig. S3C). Collectively, ChIP-seq and fluorescence microscopy data strongly suggest that while PRDE-1 primarily associates with piRNA clusters, ATTF-6 binds to both piRNA and 5S rDNA-SL1 clusters.

### ATTF-6 associates with some protein-coding genes including histone genes

**Figure 3.**
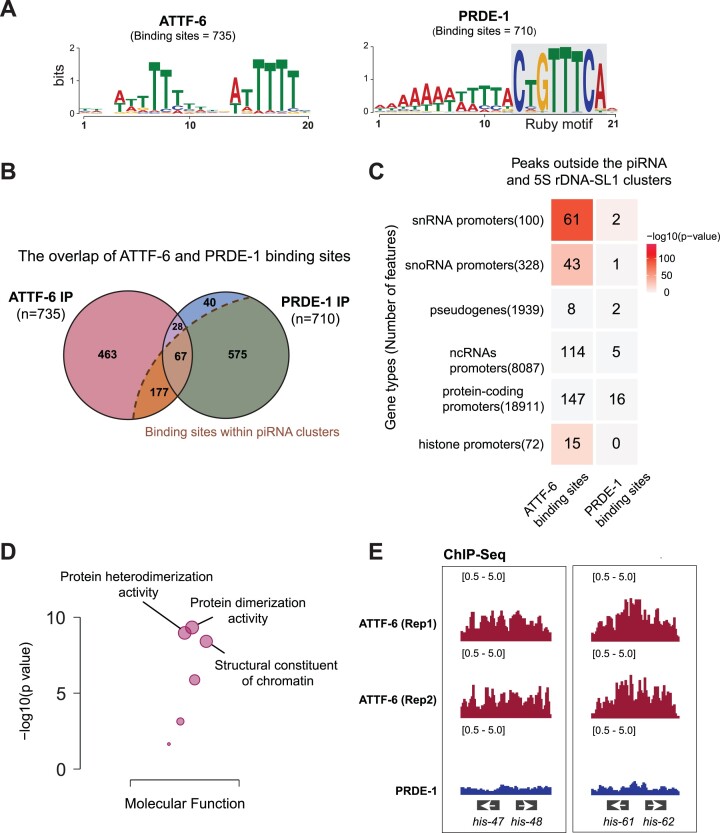
ATTF-6 associates with some protein coding genes including histone genes. (**A**) Motif analysis of ATTF-6 and PRDE-1 binding sites. The left and right panels display sequence logos for ATTF-6 and PRDE-1, respectively. 735 ATTF-6 and 710 PRDE-1 binding peaks were identified in the ChIP-Seq analysis using peak calling [47, 48]. Filtering parameters are described in the ‘Materials and methods’ section. (**B**) Venn diagram illustrating the overlap of ATTF-6 and PRDE-1 binding sites. The dashed line and dark orange region highlight the binding sites within piRNA clusters (IV: 4.75–7.5 Mb and 13.5–17.5 Mb). (**C**) ChIP-enriched signals of ATTF-6 and PRDE-1 outside piRNA and 5S rDNA-SL1 gene clusters. The heatmap represents protein binding sites overlapping with selected annotated genes, including snRNA promoters, snoRNA promoters, pseudogenes, protein-coding gene promoters, ncRNAs, and histone promoters. Numbers indicate overlaps between genes and annotated loci. The gradience represents the significance of these overlaps based on a hypergeometric test. (**D**) Gene ontology (GO) analysis of transcripts with promoters enriched in ATTF-6-IP. The y-axis represents the log_10_*P*-value, indicating the significance of the overlap between promoter-enriched transcripts and GO categories. The top three GO categories for molecular function are labeled. (**E**) Browser view of histone genes. The left panel displays the *his-47* and *his-48* loci, and the right panel shows the *his-61* and *his-62* loci. ChIP-seq data for ATTF-6 from two biological replicates (Rep1 and Rep2) are shown.

Compared to PRDE-1, ATTF-6 is more widely distributed across the genome (Fig. 2A). We therefore applied MACS2 algorithms to unbiasedly define the binding sites of PRDE-1 and ATTF-6 across the genome [47, 48]. In total, we identified 735 sites bound by ATTF-6 and 710 sites bound by PRDE-1 (Fig. 3A). As expected, Motif analysis revealed that PRDE-1 predominantly binds to the Ruby Motif (CTGTTTCA) (Fig. 3A). In contrast, ATTF-6 binding sites were most enriched for AT-rich DNA sequences (Fig. 3A). Binding sites with their start and end coordinates located within two piRNA clusters (4.75–7.5 Mb and 13.5–17.5 Mb on Chromosome IV) were classified as binding sites within piRNA clusters. By this definition, 90.4% of PRDE-1 binding sites (642/710) were confined to piRNA clusters (Fig. 3B). However, ATTF-6’s association with piRNA clusters is limited, occupying only 33.2% of its binding sites (244/735) (Fig. 3B). The overlap between ATTF-6 and PRDE-1 binding sites was minimal, with only 95 genes occupied by both factors (Fig. 3B). These findings suggest that despite their association with piRNA clusters, ATTF-6 and PRDE-1 recognize distinct DNA sequences.

While only 68 PRDE-1 binding sites were found outside the piRNA and 5S rDNA-SL1 clusters [20], 486 ATTF-6 binding sites were identified beyond these clusters (Fig. 3B and C). These binding sites were then compared with annotated genomic loci which revealed that ATTF-6 is associated with various noncoding RNA genes. Specifically, 114 annotated noncoding RNA promoters, 61 snRNA promoters and 43 snoRNA promoters, were associated with ATTF-6 (Fig. 3C). Among the 61 snRNA genes bound by ATTF-6, 14 corresponded to U1 snRNA, 17 to U2 snRNA, 5 to U4 snRNA, 9 to U5 snRNA, 1 to U6 snRNA, 1 to SM Y trans-splicing-associated snRNA, and 14 to SL2 (Spliced Leader 2) genes. Unlike SL1 genes, SL2 genes are scattered throughout the *C. elegans* genome [64] (Supplementary Fig. S4A–C). ChIP-qPCR revealed over 10-fold increase in ATTF-6 binding at the *sls-2.1* gene over the negative control (Supplementary Fig. S4D).

As anticipated for a transcription factor, ATTF-6 binding was enriched in promoter regions, mostly within 1000 nts upstream of the transcriptional start sites of protein-coding genes (Supplementary Fig. S4E). Of note, promoters of 162 protein-coding gene were associated with ATTF-6 (Fig. 3C). GO enrichment analysis was conducted to characterize these protein-coding genes [52]. The top three GO terms were ‘protein dimerization activity’, ‘protein heterodimerization activity’ and ‘structural constituent of chromatin’ (Fig. 3D). Given that all three terms include histones, we further examined histone loci, which form multiple small clusters throughout the *C. elegans* genome [6]. Indeed, ATTF-6 was enriched at the promoter regions of 15 histone genes, a pattern not observed in the PRDE-1 ChIP-seq data (Fig. 3E). In summary, our findings suggest that ATTF-6 and PRDE-1 display distinct binding patterns. While both proteins associate with piRNA clusters, ATTF-6 uniquely binds to 5S rDNA-SL1 cluster and histone clusters.

### ATTF-6 promotes the expression of histone genes, spliced leaders, and type I piRNAs

To determine whether ATTF-6 is essential for the expression of its targets, we employed (RNA interference) to deplete *attf-6* [29]. RNAi against *attf-6* led to a significant reduction in fertility, as measured by much smaller brood sizes (Supplementary Fig. S5A). Furthermore, *attf-6* RNAi-treated animals exhibited varying degrees of developmental delays, which makes it challenging to obtain a synchronized population for RNA purification. To overcome this limitation, we employed the AID (auxin-inducible degron) system to selectively deplete ATTF-6 protein in the germ line [28]. To this end, the *gfp::aid::attf-6* strain was crossed with the *mex-5p::tir1* strain in which the adaptor protein TIR1 is expressed under the germline-specific mex-5 promoter [27]. Synchronized L1 larvae were cultured without auxin until the L4 larval stage, at which point they were transferred to auxin-containing plates to induce degradation of GFP::AID::ATTF-6 (Supplementary Fig. S5B). Imaging of adult worms revealed efficient depletion of GFP::AID::ATTF-6 in the *gfp::aid::attf-6*; *mex-5p::tir1* strain (Supplementary Fig. S5C). Furthermore, we found depletion of ATTF-6 from the L4 larval stage led to germline atrophy (Supplementary Fig. S5C), suggesting ATTF-6 is required for germline development.

**Figure 4.**
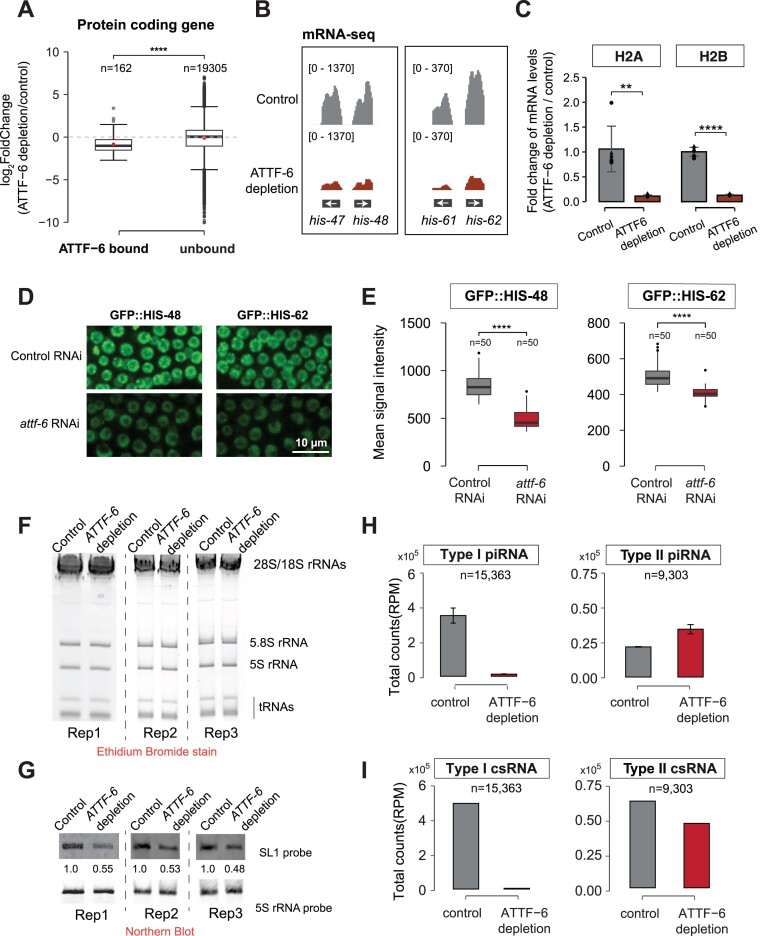
ATTF-6 promotes the expression of histone genes, spliced leader, and type I piRNAs. (**A**) Box plot showing the log_2_ fold change of transcripts from mRNA-Seq in the ATTF-6 depletion strain compared to control. Transcripts are grouped by genes with promoters bound or unbound by ATTF-6. Dots within box indicate the median value for each group. Genes with raw counts lower than 1 from mRNA-seq were excluded from this analysis. ****P< .00005, two-tailed Student’s *t*-test. (**B**) Browser tracks showing the abundance of *his-47* and *his-48* and *his-61* and *his-62* measured by mRNA sequencing in control and ATTF-6 depletion strains. Coverage represents the average of two sequencing replicates. Signals displayed on the genome browser are normalized as reads per million (RPM). (**C**) RT-qPCR analysis of H2A and H2B expression in control and ATTF-6 depletion strains. Relative expression levels of H2A and H2B were normalized to *act-3* transcripts. The bar plot shows mean ± SD from two biological replicates, each with three technical replicates. Statistical significance was determined using a two-tailed Student’s *t*-test: ‘**’ indicates *P*< .005, and ‘****’ indicates *P*< .00005. Data are presented as relative fold changes compared to the control group using the ΔΔCt method. (**D**) Maximum intensity projections of a Z-stack spanning the germline of live animals expressing GFP::HIS-48 and GFP::HIS-62, treated with control and *attf-6* RNAi. The confocal images were taken using a 40× objective. Scale bar: 10 μm. (**E**) Quantification of GFP signal intensity in germline nuclei from control and *attf-6* RNAi-treated worms. Box plots display the mean signal intensity of GFP::HIS-48 (left) and GFP::HIS-62 (right) in the nuclei. Intensity was measured in 5 independently imaged worms, with 10 randomly selected nuclei per worm (50 nuclei total per sample). ****P< .00005, two-tailed Student’s *t*-test. (**F**) Ethidium bromide stain showing the abundance of transfer RNAs (tRNAs) and rRNAs, including 5S rRNA, in control and ATTF-6 depletion strains from three biological replicates (Rep1, Rep2, and Rep3). (**G**) Northern blot displaying SL1 RNA and 5S rRNA abundance in control and ATTF-6 depletion strains. The relative abundance of SL1 RNA was normalized to that of 5S rRNA in each biological replicate. (**H**) Bar plots showing total levels of Type I and Type II piRNAs in control and ATTF-6 deletion samples. Data are presented as mean ± SD from two biological replicates. (**I**) Bar plots showing total levels of Type I and Type II csRNAs (piRNA precursors) in control and ATTF-6 deletion samples. Data represent a single biological replicate.

We purified total RNAs from *mex-5p::tir1* and *gfp::aid::attf-6; mex-5p::tir1* worms, depleted rRNAs with antisense oligonucleotides [33], and performed mRNA sequencing (mRNA-seq). Protein-coding genes were categorized as ATTF-6-bound or unbound based on ATTF-6 ChIP-seq data. Upon ATTF-6 depletion in the animal germ line, the expression levels of ATTF-6-bound genes (*n* = 162) showed a general reduction compared to the control (Supplementary Fig. S6A and Supplementary Table S4). Specifically, the median abundance of ATTF-6 targets (*n* = 162) was reduced approximately two-fold, whereas such reduction was not observed in the unbound gene category (Fig. 4A). Because ATTF-6 depletion resulted in germline atrophy (Supplementary Fig. S5C), we further assessed the expression levels of germline-expressing genes [65]. Indeed, when ATTF-6 was depleted, the median abundance of germline transcripts showed a modest 1.2-fold reduction compared to the control (Supplementary Fig. S6B).

Among ATTF-6 targets, histone mRNAs including HIS-47 (H2A), HIS-48 (H2B), HIS-61 (H2A), and HIS-62 (H2B) were markedly reduced in loss of ATTF-6 (Fig. 4B). To corroborate this mRNA-seq result, we designed primers targeting H2A and H2B transcripts and used RT-qPCR to quantify their expression. Consistent with the mRNA-seq data, the levels of both transcripts were significantly reduced upon depletion of ATTF-6 (Fig. 4C). To further assess the impact of ATTF-6 on histone expression, we measured and quantified GFP signals in strains expressing GFP::HIS-48 or GFP::HIS-62 expressed from their endogenous loci [66]. When *attf-6* was depleted using RNAi, we observed a significant reduction of GFP::HIS-48 and GFP::HIS-62 signals relative to control RNAi in the germ line (Fig. 4D and E). This decrease was also observed in intestinal cells (Supplementary Fig. S6C and D), consistent with ATTF-6 expression patterns in these tissues (Fig. 1D and Supplementary Fig. S1D).

Next, we investigated whether ATTF-6 is required for the expression of SL1. Since the mRNA-seq library prep kit is optimized to clone RNAs longer than 200-nts, SL1 which is ∼100-nts in length was not adequately cloned. Instead, we used northern blotting to quantify SL1 levels. Total RNA was subjected to denaturing gel electrophoresis and visualized using ethidium bromide, which stains abundant RNA species such as tRNAs, 5.8S, 28S, 18S, and 5S rRNAs. Depletion of ATTF-6 did not affect the levels of any of these RNAs, including 5S rRNA (Fig. 4F). Total RNAs from this denaturing gel were subsequently transferred to a positively charged nylon membrane which was probed against an SL1 probe as well as a 5S rRNA probe. Northern blot analysis from three biological replicates revealed that SL1 levels were reduced by ∼50% upon ATTF-6 depletion, after normalization to 5S rRNA (Fig. 4G and Supplementary Table S5). These findings suggest that although ATTF-6 was highly enriched at the promoter region of both 5S rDNA and SL1 genes (Fig. 2G and H), it may preferentially promote SL1 expression.

Finally, we examined whether ATTF-6 is necessary for piRNA expression. To this end, we cloned and deep sequenced small RNAs from *mex-5p::tir1* and *gfp::aid::attf-6; mex-5p::tir1* worms using a previously established small RNA cloning protocol [30–32]. The level of type I piRNAs was reduced by 29-fold upon loss of ATTF-6, while the level of type II piRNAs were modestly increased (Fig. 4H and Supplementary Table S6). To corroborate these observations, we used RT-qPCR to quantify the levels of four randomly selected and abundant piRNAs. Consistent with the small RNA-seq data, the level of all four piRNAs were strongly reduced upon depletion of ATTF-6 (Supplementary Fig. S6E). To further validate that ATTF-6 functions at the transcription step, we quantified csRNA levels using the CIP-TAP cloning followed by deep sequencing [13]. Consistent with the observed decrease in mature piRNA levels, ATTF-6 depletion led to a 120-fold reduction in type I csRNAs, with a modest effect on type II csRNAs (Fig. 4I). Taken together, our high-throughput sequencing and genetic data revealed that ATTF-6 promotes the expression of histone genes, spliced leaders, and type I piRNAs.

### AT-rich motif at piRNA promoter is required for piRNA expression

We further investigated how ATTF-6 stimulates the production of piRNAs. Based on the observation that ATTF-6 is highly enriched at multiple genomic loci within piRNA clusters and shows modest enrichment at the promoters of piRNA-producing loci (Fig. 2B–E), we developed two hypotheses: [1] Analogous to enhancers [67], ATTF-6 binding sites stimulate the expression of piRNAs from a distance; and [2] ATTF-6 directly binds to the AT-rich regions of promoters to activate piRNA transcription.

**Figure 5.**
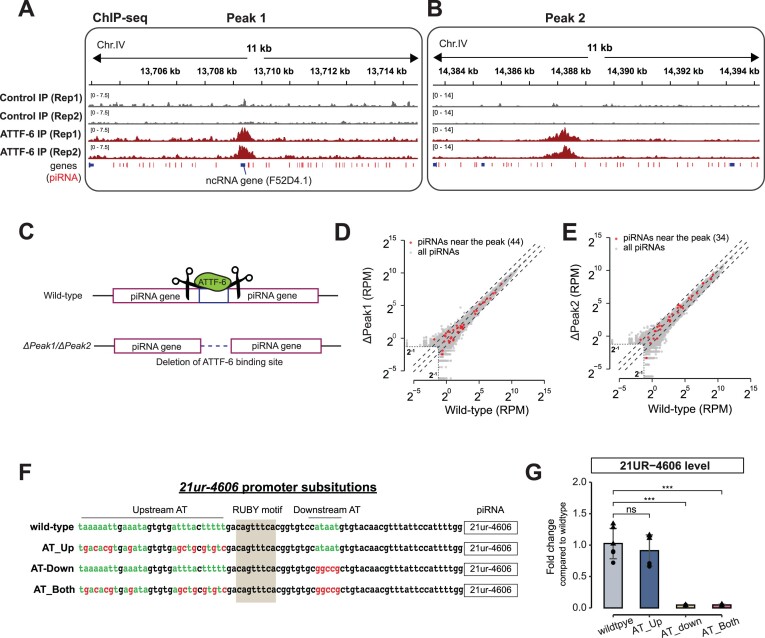
AT-rich sequences are required for piRNA expression. (A-B) Browser view of ATTF-6 binding to noncoding regions (**A**) and unannotated genomic regions (**B**). The plots display ChIP-Seq signals for Control-IP and ATTF-6-IP across the peak binding region. piRNA genes surrounding the the peak are marked by red lines. In panel (A), the ncRNA gene F52D4.1, overlapping with peak 1, is labeled. ChIP-seq data for control and ATTF-6 from two biological replicates (Rep1 and Rep2) are shown. (**C**) Schematic illustrating the deletion of ATTF-6 binding sites. *ΔPeak1* represents the deleted genomic region located on chromosome IV, spanning positions 13 709 294–13 709 476 bp. *ΔPeak2* corresponds the deleted region on chromosome IV, spanning positions 14 387 943–14 388 536 bp. (**D, E**) Scatterplot showing piRNA levels in WT and ATTF-6 binding site deletion mutants: (**D**) ΔPeak1 and (**E**) ΔPeak2. Red dots represent piRNAs within 5 kb upstream and downstream of the peak summit. Top and bottom dashed lines indicate a 4-fold change. Reads below 2^–1^ RPM were excluded as background noise. (**F**) Schematic showing the promoter sequence of *21ur-4606* in WT and AT mutants. AT nucleotides upstream, downstream, or both upstream and downstream of the Ruby motif were substituted with CG nucleotides to disrupt the AT-rich sequence. *AT_Up* refers to upstream mutations, *AT_Down* to downstream mutations, and *AT-Both* to mutations in both regions. (**G**) RT-qPCR analysis of 21UR-4606 expression in WT and promoter AT substitution mutants. Relative levels of 21ur-4606 were normalized to the control 21UR-1769. The bar plot shows mean ± SD from two biological replicates, each with three technical replicates. ns, not significant, ***P< .0005, two-tailed Student’s *t*-test. Data are presented as relative fold change using the ΔΔCt method.

To test the first hypothesis, we employed CRISPR/Cas9 genome editing to remove two prominent ATTF-6 binding sites within piRNA clusters, each surrounded by dozens of piRNA-producing loci (Fig. 5A and B). One ATTF-6 binding site overlapped with the noncoding RNA gene F52D4.1 (Figure 5A), while the other lacked any annotated genes aside from a few piRNA genes (Fig. 5B). Two pairs of CRISPR guide RNAs were used to delete the peaks of these two ATTF-6 binding sites (Fig. 5C). Specifically, one pair targeted and removed the region between positions 13 709 294 and 13 709 476 on Chromosome IV (*ΔPeak1*), and the other pair deleted the region between positions 14 387 943 and 14 388 536 on Chromosome IV (*ΔPeak2*). We then cloned and deep-sequenced small RNAs isolated from mutants with individual ATTF-6 binding sites deleted. Compared to wild-type (WT) strain, neither global piRNA expression nor piRNAs adjacent to ATTF-6 peaks were significantly altered in these two mutant strains (Fig. 5D and E). These findings indicate that ATTF-6 is unlikely to stimulate piRNA expression from a distance.

To test the second hypothesis that AT-rich sequences promote piRNA transcriptiong, we mutated the promoter of a single piRNA gene, *21ur-4606*, which produces an abundant piRNA. Consistent with consensus sequences of piRNA-producing loci (Supplementary Fig. S1A and B), the *21ur-4606* gene contains the Ruby motif, as well as upstream and downstream AT-rich sequences (Fig. 5F). Using CRISPR/Cas9 genome editing, we generated three mutant strains in which the upstream A/T, downstream A/T, and both A/T sequences were converted into G/C sequences (Fig. 5F). We then purified total RNAs and quantified 21UR-4606 level using RT-qPCR. After normalized to an unrelated piRNA, 21UR-1769, the upstream AT mutant showed a modest decrease in 21UR-4606 expression relative to WT (Fig. 5G). However, mutations in the downstream AT-rich and both AT-rich sequences led to over 21-fold reduction in the 21UR-4606 level (Fig. 5G). To assess the impact of AT-rich sequence mutations on ATTF-6 binding, we performed ChIP-seq on the strain containing mutations in both upstream and downstream AT-rich sequences of *21ur-4606*. As a positive control, the SL2 gene displayed robust ATTF-6 ChIP signals in both WT and AT sequence mutant animals (Supplementary Fig. S7A). However, coverage at 21UR-4606 was too low to definitively determine whether the AT motif mutations affected ATTF-6 binding (Supplementary Fig. S7B). We conclude that possibly through binding to ATTF-6, the AT-rich motif at piRNA promoter is required for piRNA expression.

### ATTF-6 is required for the recruitment of USTC to the piRNA clusters

**Figure 6.**
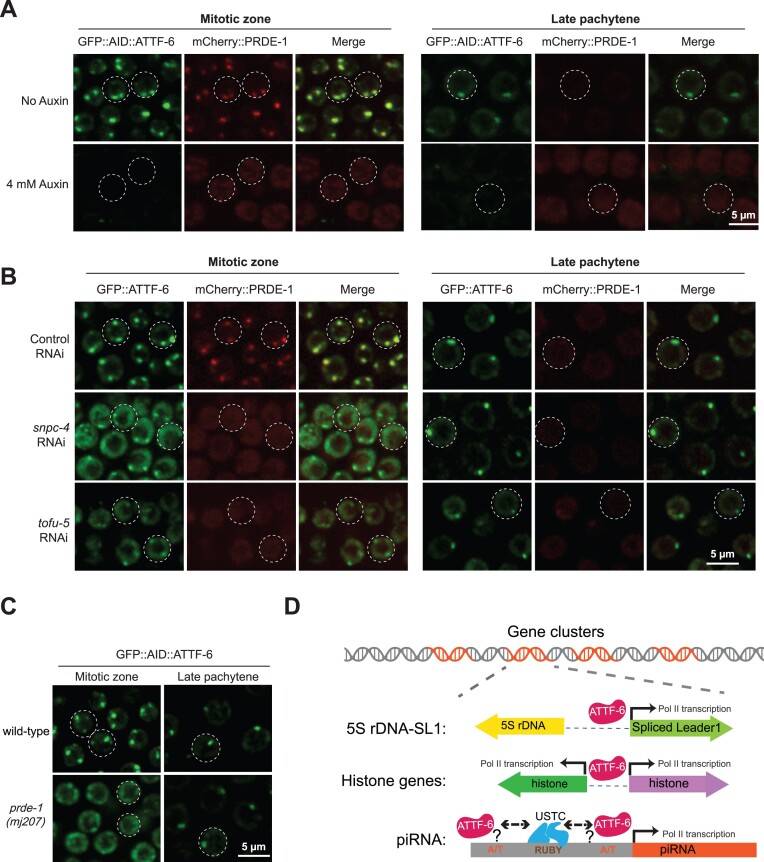
ATTF-6 is required for the recruitment of USTC to piRNA clusters. (**A–C**) Maximum intensity projections of a Z-stack spanning the germline of live animals, showing fluorescently tagged proteins localized to the mitotic zone and late pachytene region. (**A**) Expression of GFP::AID::ATTF-6 and mCherry::PRDE-1 with or without 4 mM auxin treatment. (**B**) Expression of GFP::AID::ATTF-6 and mCherry::PRDE-1 following control RNAi, *snpc-4* RNAi, and *tofu-6* RNAi treatments. (**C**) Expression of GFP::AID::ATTF-6 in the mitotic zone and late pachytene region in WT and *prde-1 (mj207)* mutant. Each dotted circle indicates a germline nucleus. The confocal images were taken using a 60× objective. Scale bar: 5 μm. (**D**) Model illustrating the proposed role of ATTF-6 in clustered gene transcription.

We performed a series of experiments to investigate the sequential order and co-dependency of ATTF-6 and USTC component binding to piRNA clusters. First, we employed a strain expressing GFP::AID::ATTF-6, mCherry::PRDE-1 and germline TIR1. In the absence of auxin, GFP::AID::ATTF-6 and mCherry::PRDE-1 co-localized at piRNA clusters in the mitotic zone (Figs 1E and 6A). As expected, auxin treatment led to the depletion of ATTF-6 in both mitotic and pachytene nuclei (Fig. 6A). Following ATTF-6 depletion, PRDE-1 became diffuse and failed to form distinct foci presumably corresponding to piRNA clusters (Fig. 6A). This observation suggests that the formation of PRDE-1 foci at piRNA clusters requires ATTF-6.

Next, we performed reciprocal experiments to determine whether the formation of ATTF-6 foci depends on USTC components. We employed RNAi to deplete additional USTC component, including SNPC-4 and TOFU-5, from the *gfp::aid::attf-6*; *mCherry::prde-1* strain. Depletion of either SNPC-4 or TOFU-5 led to the diffusion of mCherry::PRDE-1 and loss of large ATTF-6 foci (Fig. 6B). However, small ATTF-6 foci, which are associated with the 5S rDNA-SL1 cluster, were still readily detected in the late pachytene region of *snpc-4* or *tofu-5* RNAi-treated samples (Fig. 6B). Additionally, by crossing the GFP::AID::ATTF-6 strain with *prde-1 (mj207)* mutants [17], we found that GFP::AID::ATTF-6 failed to form large piRNA foci in mitotic region (Fig. 6C). Similar to the observation from *snpc-4* or *tofu-5* RNAi, *prde-1* mutation did not appear to affect the formation of small ATTF-6 foci at the 5S rDNA-SL1 cluster in the late pachytene region (Fig. 6C). Taken together, these results suggest that the binding of ATTF-6 and USTC components to piRNA clusters is co-dependent, while the binding of ATTF-6 to the 5S rDNA-SL1 cluster occurs largely independently of USTC components.

## Discussion

Recent advances in genomics and transcriptomics have identified many gene clusters across all three domains of life. Despite these discoveries, considerable knowledge gaps remain, particularly regarding the regulation and chromatin organization of gene clusters. Key questions include: How are gene clusters regulated at the transcriptional level? Which specific transcription factors and regulatory proteins target and modulate these gene clusters? In this study, using a proximity labeling approach with *C. elegans* TurboID::PRDE-1, we uncovered ATTF-6 as a novel factor that forms distinct nuclear foci and associates with multiple gene clusters. Unlike PRDE-1 which primarily associates with piRNA-producing clusters, ATTF-6 binds to a broader range of clusters, including piRNA, 5S rDNA-SL1, and histone clusters (Fig. 6D). Our findings demonstrate that ATTF-6 is essential for the proper expression of piRNAs, spliced leaders, and histones, potentially through its interaction with AT-rich sequences in their promoters or through its interaction with other chromatin factors. This work provides insights into the mechanisms governing the transcriptional regulation of clustered genes in *C. elegans* and facilitates future studies exploring the broader roles of AT-hook proteins in gene regulation in other organisms.

### Mechanism of ATTF-6 action

AT-hook transcription factors are characterized by the presence of AT-hook motifs—short peptide sequences (glycine–arginine–proline) that bind to the minor groove of AT-rich DNA sequences [24]. These transcription factors are found across eukaryotes and play fundamental roles in regulating gene expression and chromatin architecture. By binding to AT-rich regions, AT-hook transcription factors can alter the conformation of chromatin, making it more or less accessible to other transcription factors and RNA polymerases and modulating the transcription of target genes [24, 62, 63].

Our study showed that ATTF-6, similar to other AT-hook containing proteins, binds to AT-rich sequences (Fig. 3A). However, ATTF-6 is unique in its association with piRNA, 5S rDNA-SL1, and histone clusters (Fig. 6D). In general, ATTF-6 stimulates the expression of target genes, though it does so via different mechanisms. For example, at piRNA clusters ATTF-6 is required for the recruitment of PRDE-1 to piRNA-producing loci, possibly by increasing the accessibility of piRNA promoters which enables the binding of USTC. At the 5S rDNA-SL1 cluster, ATTF-6 acts independent of PRDE-1 to promote gene expression (Fig. 6D). ATTF-6 is highly enriched at the promoter region of 5S rDNA and SL1 genes (Fig. 2H and I). Notably, it is required for the accumulation of SL1, but not mature 5S rRNA (Fig. 4F and G). This observation indicates that ATTF-6 may preferentially stimulate RNA Pol II-mediated transcription over RNA Pol III-mediated transcription. However, given the complexity of rRNA biogenesis [68], further studies, including the determination of RNA Pol II and RNA Pol III occupancies at the 5S rDNA-SL1 cluster and the quantification of transcription rates for 5S rDNA and SL1 genes in WT and *attf-6* depletion backgrounds, will be required to test this hypothesis. A previous study demonstrated that TOFU-5 plays a role in starvation-induced SL1 RNA synthesis [69]. Future work could explore the involvement of ATTF-6 in SL1 transcription under different stress conditions. Finally, since the mechanism by which ATTF-6 induces histone expression remains unclear, further investigation is needed to identify other transcription factors that cooperate with ATTF-6 to promote the transcription of histone clusters.

This study does have limitations regarding the mechanism of ATTF-6 action. First, since AT-rich motifs are in close proximity to the Ruby motif, ChIP-seq did not provide sufficient resolution to distinguish ATTF-6 binding at these neighboring sites. Future work could overcome this limitation by employing ChIP-exo, which offers higher resolution for binding site analysis [70]. Second, the specific roles of the AT-hooks and other domains in ATTF-6 were not fully characterized. Questions such as whether AT-hooks make chromatin more accessible, or if other domains interact with components of the RNA polymerase II transcription machinery, remain to be explored. Future investigations, incorporating biochemical assays, will be necessary to address these questions. Despite these limitations, the discovery of ATTF-6’s involvement in organizing specific nuclear foci underscores the complexity of transcriptional regulation in germ cells and opens new avenues for understanding how AT-hook transcription factors modulate the expression of clustered genes.

### Proximity labeling for identifying chromatin factors in gene clusters

Proximity labeling, combined with mass spectrometry, is a robust approach to study protein-protein interactions and define the local proteome in living cells [54]. One significant advantage of proximity labeling is its ability to capture weak and transient interactions that might be missed using other methods. Here we employed proximity labeling to identify proteins associated with piRNA clusters. The TurboID::PRDE-1 fusion protein successfully labeled all of known piRNA chromatin factors, including USTC components and SNPC proteins. Importantly, it also identified additional factors that are not been characterized in the context of the piRNA pathway, such as ATTF-6 (Fig. 1A and B). Interestingly, immunoprecipitation of USTC components including PRDE-1 and SNPC-4 followed by mass spectrometry did not enrich ATTF-6 [20, 58], suggesting ATTF-6 is not in a stable complex with, or does not directly interact with USTC. This result highlights the unique capability of proximity labeling to reveal interactors that are part of the same functional network but not directly bound to the target protein.

While this study focused on characterizing ATTF-6, many other factors identified through proximity labeling remain to be explored. The mass spectrometry dataset generated here offers a valuable resource for the *C. elegans* and piRNA research communities. Beyond this, the approach developed in our study could be applied to define chromatin factors in other gene clusters in *C. elegans* and other organisms.

## Supplementary Material

gkaf079_Supplemental_Files

## Data Availability

ChIP-Seq data have been deposited at NCBI under the accession number GSE277641 (https://www.ncbi.nlm.nih.gov/geo/query/acc.cgi?acc=GSE277641). mRNA-Seq data have been deposited under the accession number GSE277642 (https://www.ncbi.nlm.nih.gov/geo/query/acc.cgi?acc=GSE277642). Small RNA-Seq data have been deposited under the accession number GSE277643 (https://www.ncbi.nlm.nih.gov/geo/query/acc.cgi?acc=GSE277643). PRDE-1 ChIP-seq data have been deposited at NCBI under the accession number GSE112682 [20]. Raw mass spectrometry data along with result files have been deposited to the ProteomeXchange Consortium via the PRIDE partner repository [71] with the dataset identifier PXD055895 and project DOI: 10.6019/PXD055895.
