# Supplementary material for: An AT-hook transcription factor promotes transcription of histone, spliced-leader, and piRNA clusters
Source: Nucleic Acids Res. 2025 Feb 13;53(4):gkaf079. doi: 10.1093/nar/gkaf079 (PMC11822377; doi:10.1093/nar/gkaf079)
Supplement: gkaf079_Supplemental_Files [file gkaf079_supplemental_files.zip › 2_supplementary legends and captions.docx]

**Supplementary Figure 1: piRNA-producing loci and expression of ATTF-6.**

(**A-B**) Consensus sequences of piRNA-producing genes in *C. elegans* (A) and *C. briggsae* (B). The nucleotide composition is shown for 60 nucleotides upstream of annotated piRNA genes.

(**C-D**) GFP::AID::ATTF-6 expression in somatic cells. Confocal images (60x objective) represent GFP::AID::ATTF-6 localization in somatic nuclei, including body wall muscle (C) and intestine nuclei (D). The images are maximum intensity projections of a Z-stack spanning the somatic nuclei. Scale bar: 10 μm.

**Supplementary Figure 2: Expression of PRDE-1 and ATTF-6.**

(**A**) Maximum intensity projection of a Z-stack spanning the germ nuclei of fixed adult worms expressing GFP::AID::ATTF-6 and mCherry::PRDE-1. Germ nuclei in the mitotic zone, early-mid pachytene, and late pachytene are shown. Arrowheads indicate small foci that do not colocalize with mCherry::PRDE-1 foci. Scale bar: 5 μm.

(**B**) GFP::PRDE-1 expression in the gonad. Confocal images (60x objective) show GFP::PRDE-1 localization in germ cells. The images are maximum intensity projections of a Z-stack spanning the germline. Scale bar: 25 μm. Dashed lines outline the gonad region, with the mitotic zone, early pachytene, and late pachytene labeled.

(**C**) Quantification of GFP::PRDE-1 and mCherry::PRDE-1 foci numbers in germ nuclei. GFP::PRDE-1 foci were counted in fixed worms, and mCherry::PRDE-1 foci were counted in live animals. Foci in 10 nuclei from each germline zone were examined. Error bars represent the standard deviation (SD).

(**D**) ATTF-6::GFP expression in the gonad. Confocal images (60x objective) showing ATTF-6::GFP localization in germ cells. The images are maximum intensity projections of a Z-stack spanning the germline. Scale bar: 25 μm.

(**E**) Quantification of foci numbers in germ nuclei. 10 nuclei from each germline zone were selected to count the number of ATTF-6::GFP foci. Results are compared to the GFP::AID::ATTF-6 strain.

**Supplementary Figure 3: ChIP-seq and immunofluorescence analyses of ATTF-6.**

(**A**) Metaplot and heatmap of ATTF-6 ChIP-seq binding profiles from two biological replicates around Type I piRNA genes. The plots are anchored at the 5’ end of the Type I piRNA genes and span 1 kb upstream and downstream. Signals were calculated as ratios relative to the control-IP.

(**B**) ChIP-qPCR analysis of ATTF-6-IP and control-IP samples using primers targeting the promoter of the *21ur-2675* gene. Data represent the mean ± standard deviation (SD) from two biological replicates, each with three technical replicates.

(**C**) Localization of the 5S rDNA-SL1 gene cluster, piRNA gene cluster, and ATTF-6 in germ nuclei, including the mitotic zone, early-mid pachytene, and late pachytene. FISH probes targeting the 5S rDNA and piRNA gene clusters are listed in Supplementary Table S2. FISH was followed by immunolocalization of ATTF-6::3xFlag using an anti-Flag antibody. Arrowheads indicate the co-localization of ATTF-6 and piRNA clusters. Arrows indicate the co-localization of ATTF-6 and 5S rDNA-SL1 cluster. The confocal images (60x objective) are maximum intensity projections of Z-stacks spanning the fixed germline. Scale bar: 2 μm.

**Supplementary Figure 4: ATTF-6 binds to SL2 genes and protein-coding genes .**

(**A-C**) Browser view of ChIP signals on several SL2 genes: sls-2.1 (A), sls-2.2 (B), and sls-2.10 (C). ChIP-seq data for ATTF-6 from two biological replicates (Rep1 and Rep2) are shown.

(**D**) ChIP-qPCR analysis of ATTF-6-IP and control-IP samples using primers targeting the promoter of the sls-2.1 snRNA gene. Data represent the mean ± standard deviation (SD) from two biological replicates, each with three technical replicates.

(**E**) Metaplot and heatmap of ATTF-6 ChIP-seq binding profiles from two biological replicates around the transcription start sites (TSSs) of protein-coding genes. The plots are anchored at the TSSs and span 1 kb upstream and downstream. Signals were calculated as ratios relative to the control-IP.

**Supplementary Figure 5: Depletion of ATTF-6 using RNAi and degron system.**

(**A**) Brood size analysis of N2 worms fed with L4440 control RNAi or attf-6 RNAi. A single L1 worm was placed on each RNAi plate, and the total progeny were counted. Ten independent RNAi plates were prepared for each condition. Statistical significance was determined using a two-tailed Student’s t-test (****p < 0.00005).

(**B**) Schematic showing the design of ATTF-6 depletion using the degron system. The upper panel displays the gene constructs of the control and ATTF-6 depletion strains. The bottom panel illustrates the timeline of auxin treatment for RNA extraction.

(**C**) Maximum intensity projections of a Z-stack spanning the germline of live animals expressing GFP::AID::ATTF-6, treated without or with 4 mM auxin. The confocal images were taken using a 60x objective. Scale bar: 25 μm.

**Supplementary Figure 6: ATTF-6 promotes the expression of its targets.**

(**A**) Scatter plot showing transcript levels of protein-coding genes in the ATTF-6 depletion strain compared to the control. Protein-coding gene promoters bound by ATTF-6 are highlighted in blue. RPM (Reads Per Million) represents the average of two sequencing replicates. Genes with raw counts below 1 from mRNA-seq were excluded from this analysis.

(**B**) Box plot showing the log2 fold change of transcripts from mRNA-seq in the ATTF-6 depletion strain compared to the control strain. Transcripts are categorized as germline transcripts or non-germline transcripts (others) as defined by Ortiz et al. (65). Red dot indicates the median value for each group. Genes with raw counts below 1 from mRNA-seq were excluded from this analysis. ****p < 0.00005, two-tailed Student’s t-test.

(**C**) Maximum intensity projections of a Z-stack spanning the somatic nuclei of live animals expressing GFP::HIS-48 and GFP::HIS-62, treated with control (L4440) and attf-6 RNAi. The confocal images were taken using a 40x objective. Scale bar: 25 μm.

(**D**) Quantification of GFP signal intensity in somatic nuclei from L4440 control and *attf-6* RNAi-treated worms. Box plots display the mean signal intensity of GFP::HIS-48 (left) and GFP::HIS-62 (right) in the nuclei. Intensity was measured in 5 independently imaged worms, with 10 randomly selected nuclei per worm (50 nuclei total per sample). ****p<0.00005, two-tailed Student’s t-test.

(**E**) RT-qPCR analysis of piRNA expression in control and ATTF-6 depletion strains. The relative levels of piRNAs (21UR-1496, 21UR-3442, 21UR-4606, and 21UR-5665) were quantified and normalized to miRNA-52. The bar plot represents the mean ± standard deviation (SD) from two biological replicates, each with three technical replicates. **p<0.005, ***p<0.0005, **p<0.00005, two-tailed Student’s t-test. Data are expressed as relative fold change using the ∆∆Ct method.

**Supplementary Figure 7: Browser view of ATTF-6 ChIP at SL2 and *21ur-4606* loci.**

(**A-B**). Browser view of ATTF-6 ChIP signals for the *sls-2.1* gene (A) and the promoter region of the *21ur-4606* piRNA gene (B). The plots show ATTF-6 binding signals in wild-type and AT-rich sequence substitution (AT_Both) strains. ChIP-Seq reads are normalized to the internal 5S-rDNA-SL1 cluster and multiplied by 100. In (B), the upstream and downstream AT to GC mutation regions are highlighted in green, and the Ruby motif is highlighted in brown.

**Supplementary Table S1**: List of strains.

**Supplementary Table S2:** List of RNA and DNA oligonucleotides.

**Supplementary Table S3:** Normalized spectral counts of proteins significantly enriched from the PRDE-1 TurboID experiment.

**Supplementary Table S4:** mRNA levels in control and ATTF-6 depletion strains.

**Supplementary Table S5:** Raw image files of Ethidium bromide stain and Northern Blots.

**Supplementary Table S6:** piRNA levels in control and ATTF-6 depletion strains.
