# Supplementary figures and images for: An AT-hook transcription factor promotes transcription of histone, spliced-leader, and piRNA clusters
Source: Nucleic Acids Res. 2025 Feb 13;53(4):gkaf079. doi: 10.1093/nar/gkaf079 (PMC11822377; doi:10.1093/nar/gkaf079)

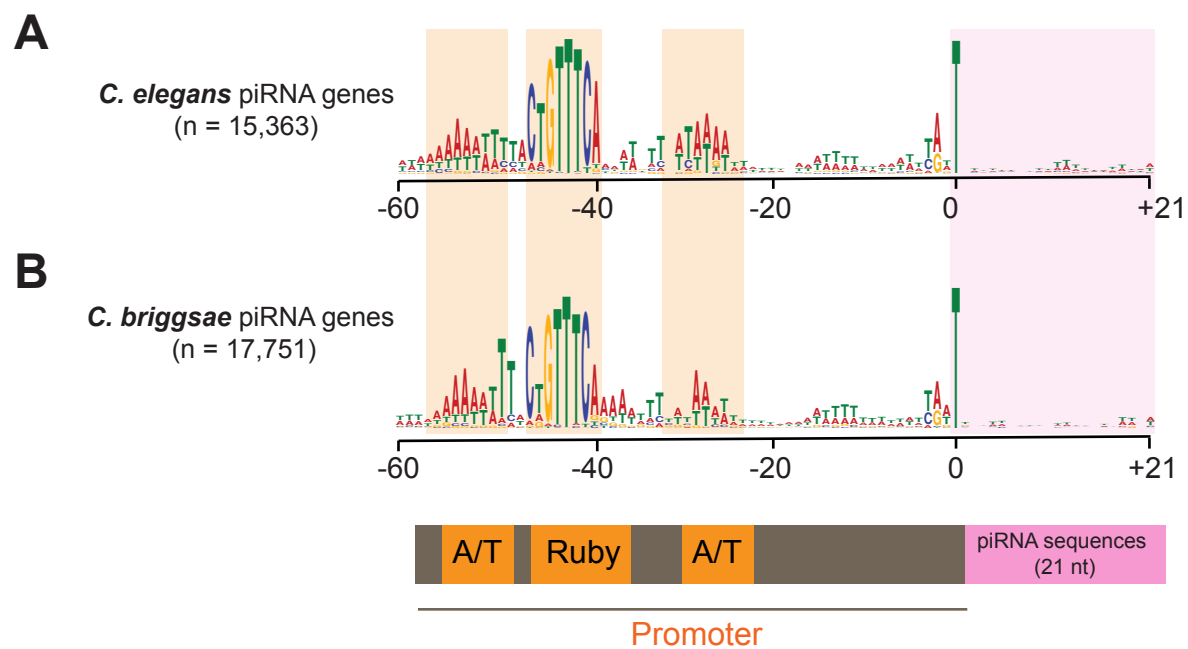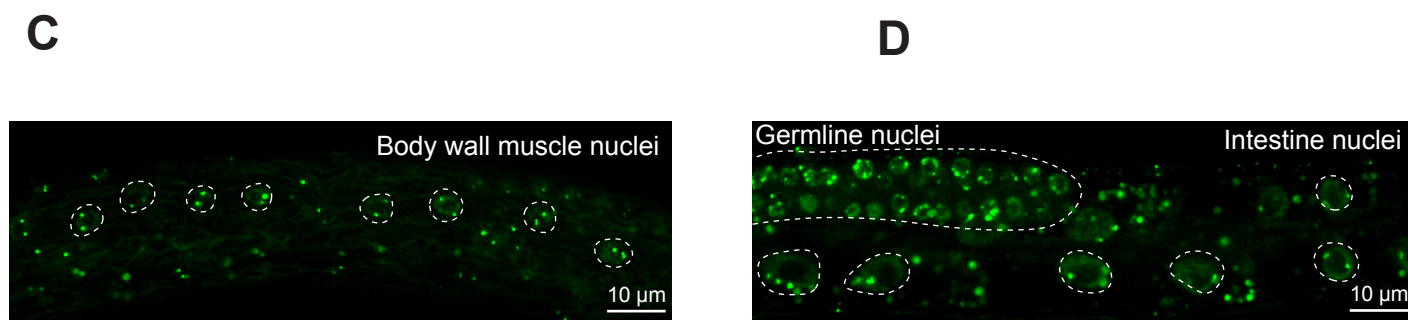

**Figure S1**

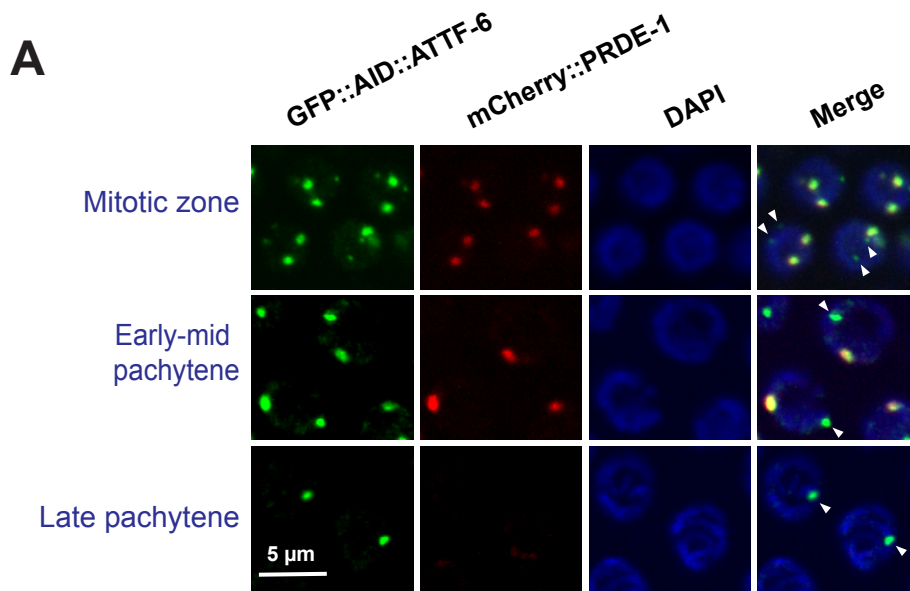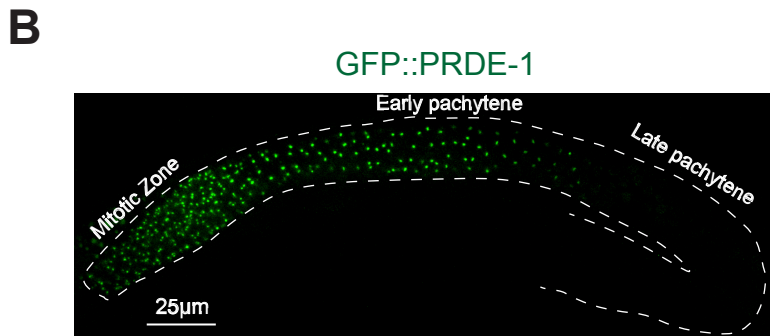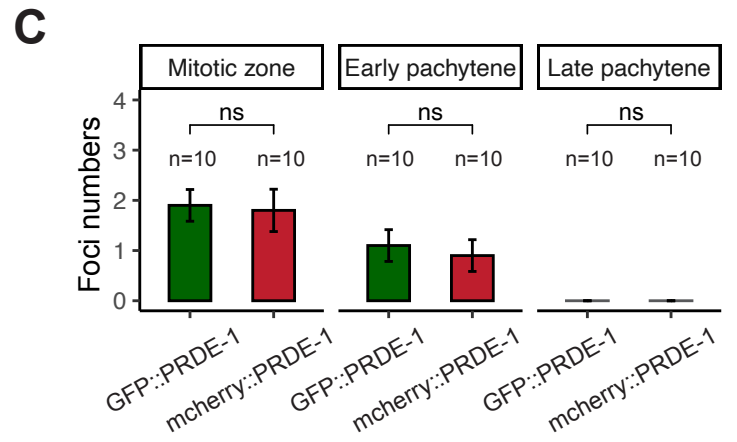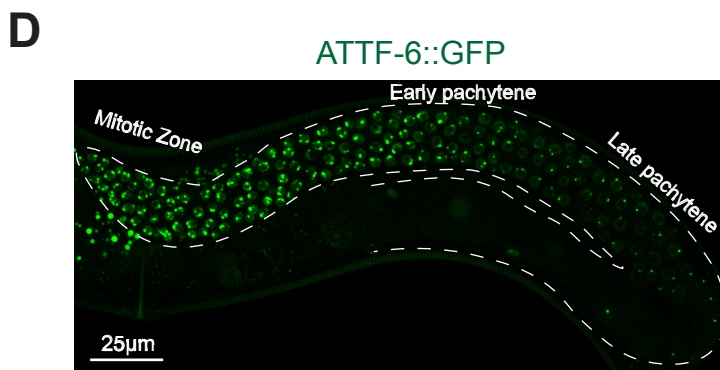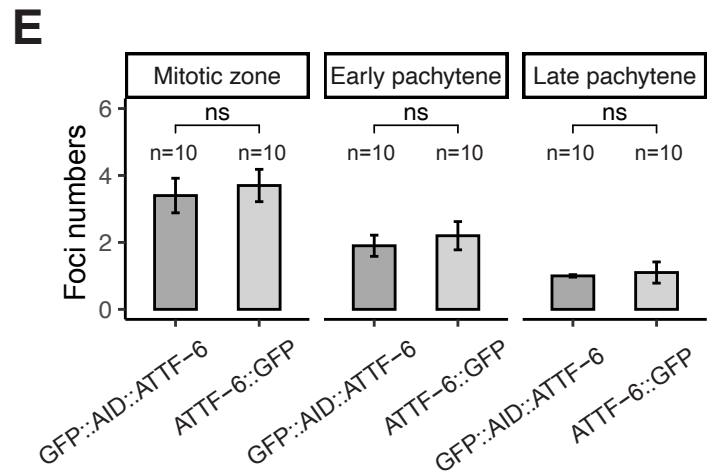

**Figure S2**

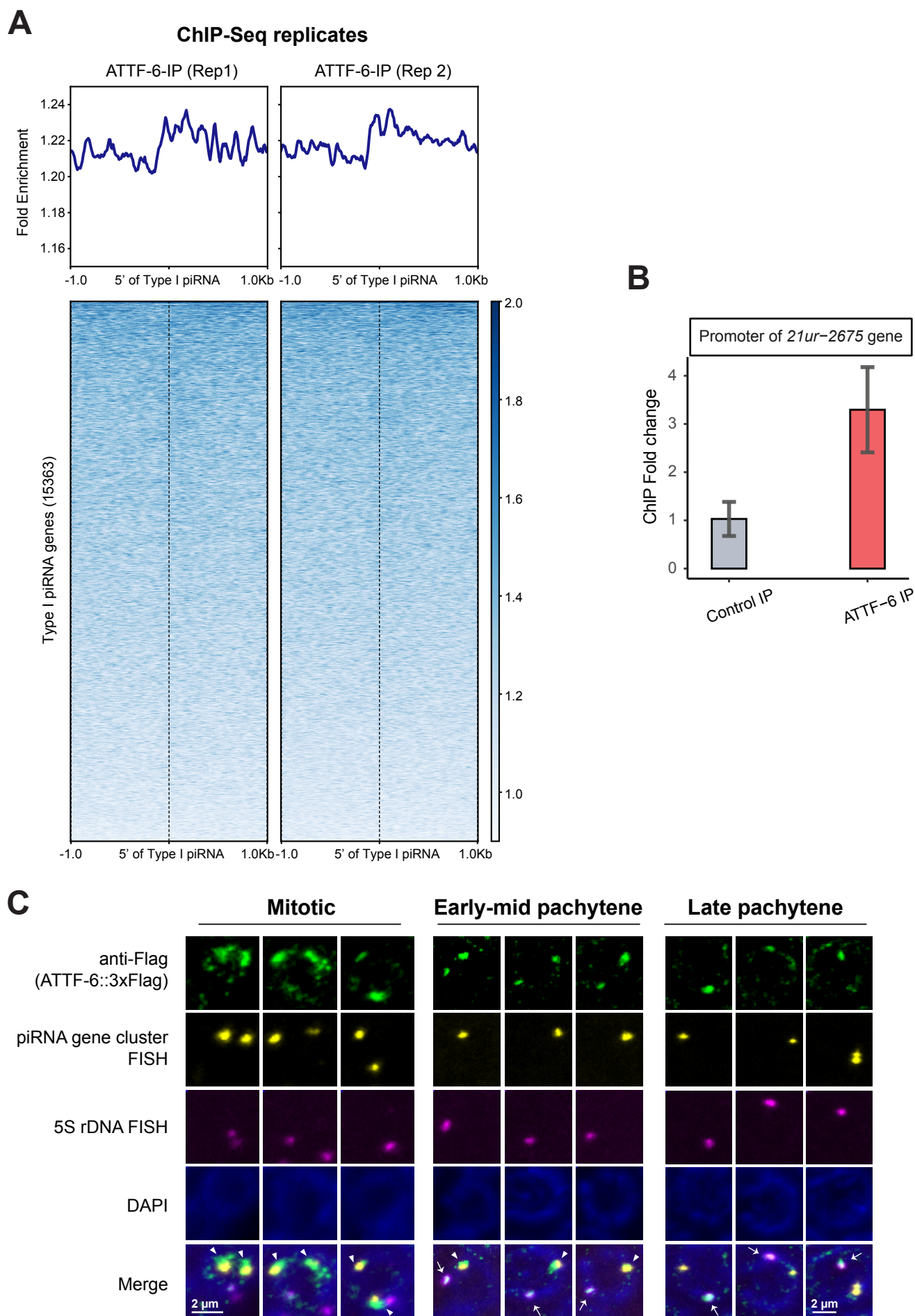

**Figure S3**

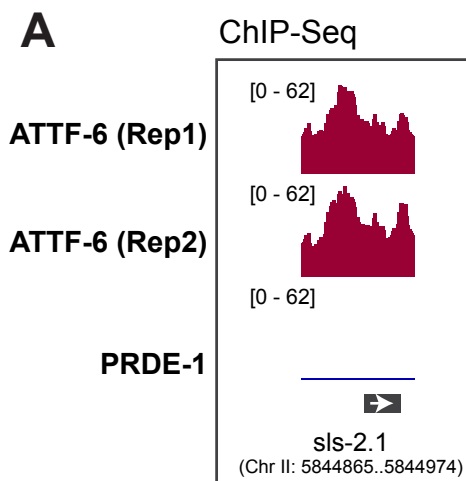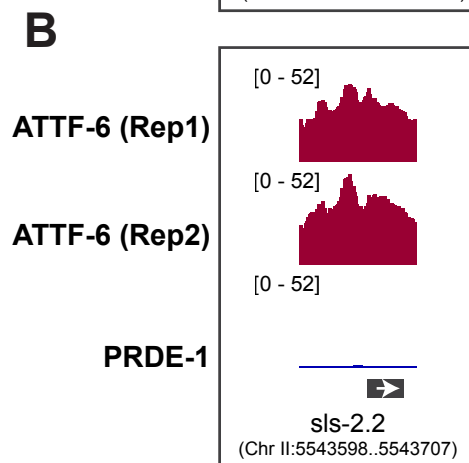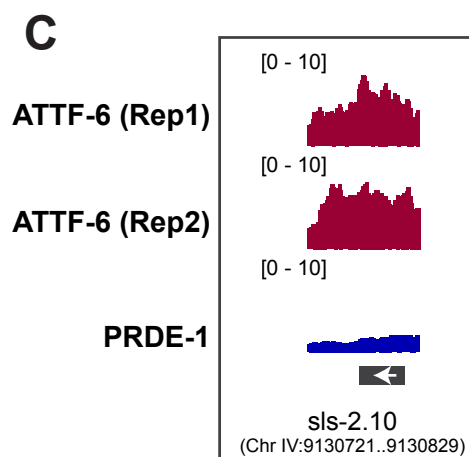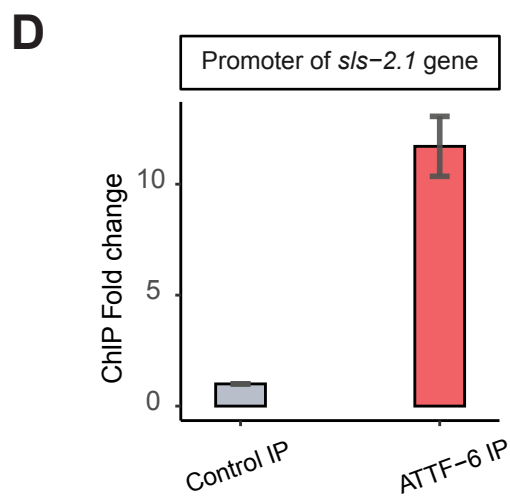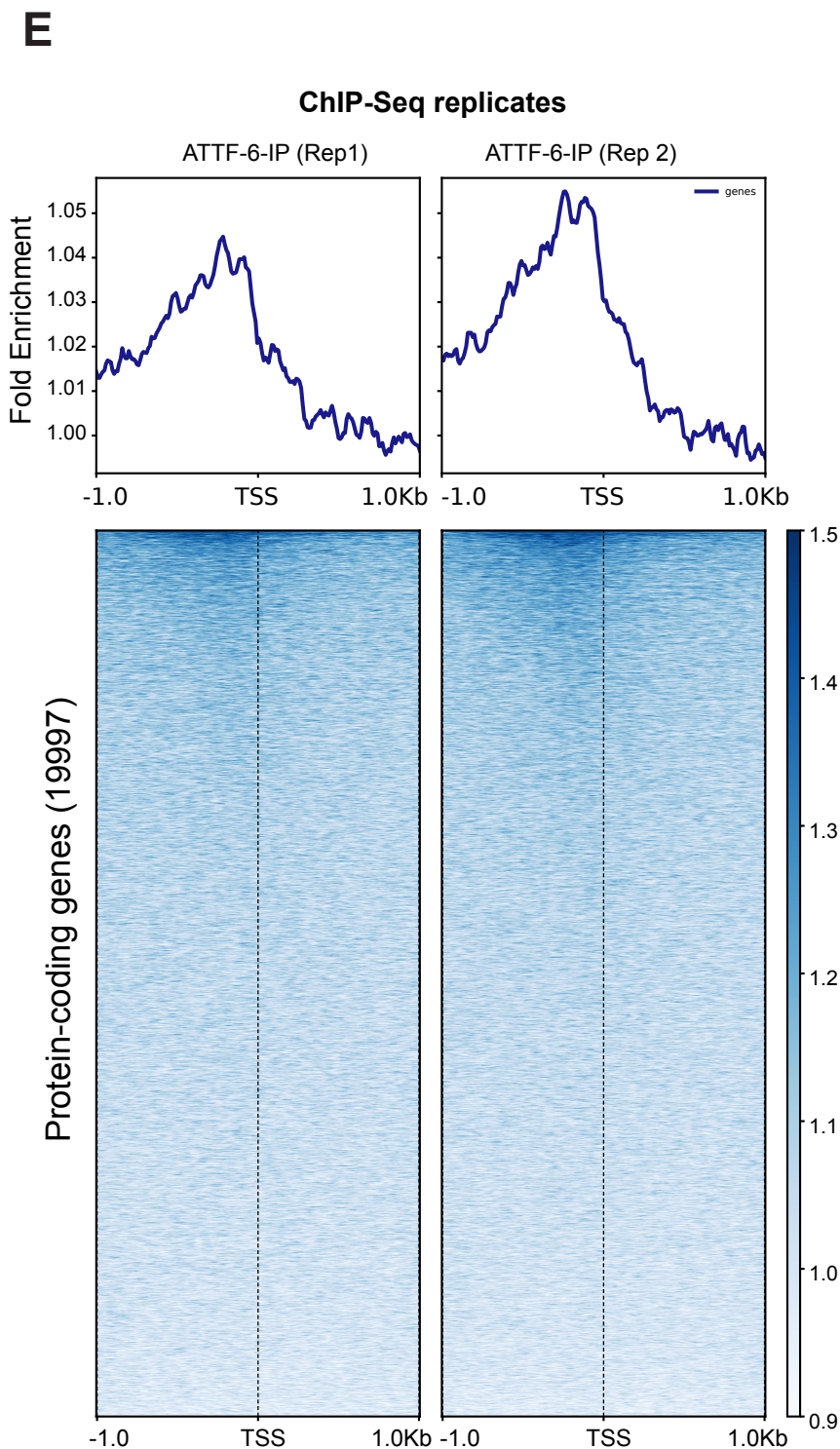

**Figure S4**

**A**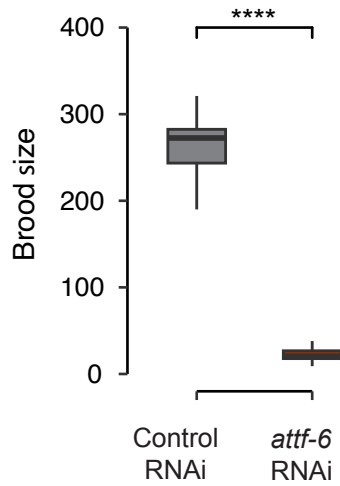**B**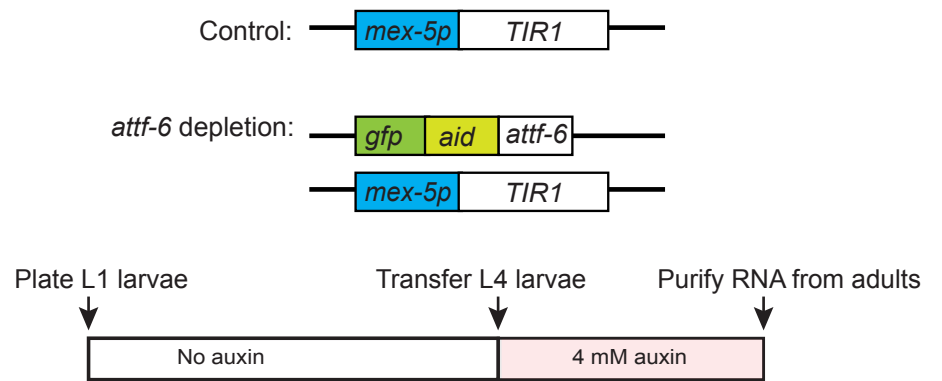**C**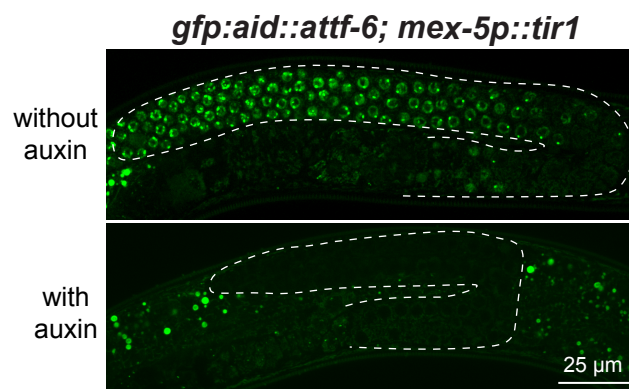**Figure S5**

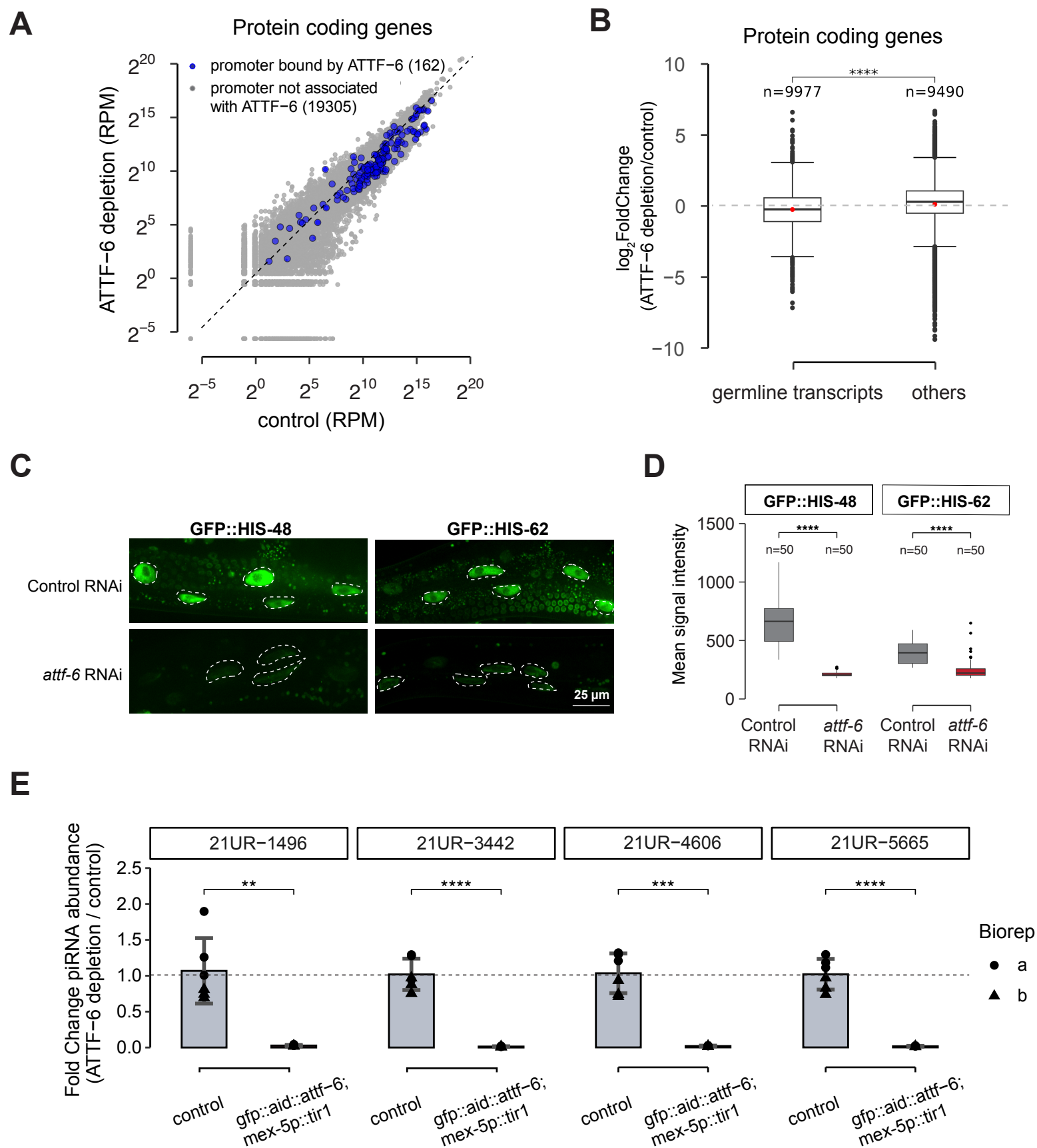

**Figure S6**

A

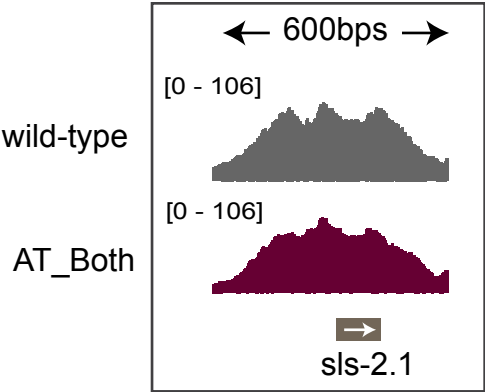

B

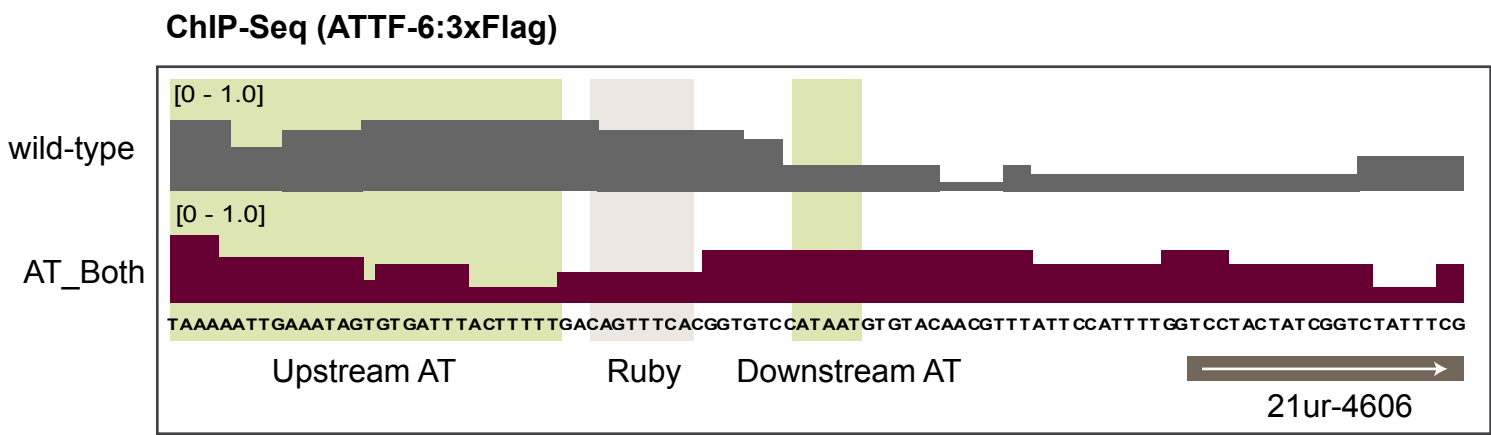

Figure S7

Supplement: gkaf079_Supplemental_Files [file gkaf079_supplemental_files.zip › Supplementary_Figure_revision.pdf]
